# Experimental and computational analyses reveal that environmental restrictions shape HIV-1 spread in 3D cultures

**DOI:** 10.1038/s41467-019-09879-3

**Published:** 2019-05-13

**Authors:** Andrea Imle, Peter Kumberger, Nikolas D. Schnellbächer, Jana Fehr, Paola Carrillo-Bustamante, Janez Ales, Philip Schmidt, Christian Ritter, William J. Godinez, Barbara Müller, Karl Rohr, Fred A. Hamprecht, Ulrich S. Schwarz, Frederik Graw, Oliver T. Fackler

**Affiliations:** 1 Department of Infectious Diseases, Centre for Integrative Infectious Disease Research (CIID), Integrative Virology, University Hospital Heidelberg, 69120 Heidelberg, Germany; 2 Centre for Modelling and Simulation in the Biosciences, BioQuant, Heidelberg University, 69120 Heidelberg, Germany; 3 Institute for Theoretical Physics and BioQuant, Heidelberg University, 69120 Heidelberg, Germany; 4 HCI/IWR, Heidelberg University, 69120 Heidelberg, Germany; 5 Biomedical Computer Vision Group, BioQuant, IPMB, and DKFZ, Heidelberg University, 69120 Heidelberg, Germany; 6 Department of Infectious Diseases, Centre for Integrative Infectious Disease Research (CIID), Virology, University Hospital Heidelberg, 69120 Heidelberg, Germany; 7 German Centre for Infection Research (DZIF), Partner Site Heidelberg, Heidelberg, Germany; 8 Present Address: Cell Biology and Biophysics Unit, European Molecular Biology Laboratory, 69117 Heidelberg, Germany; 9 Present Address: Digital Health & Machine Learning, Hasso-Plattner Institute, 14482 Potsdam, Germany; 10 Present Address: Vector Biology Unit, Max-Planck Institute for Infection Biology, 10117 Berlin, Germany

**Keywords:** Amoeboid migration, Computational models, Viral transmission, HIV infections

## Abstract

Pathogens face varying microenvironments in vivo, but suitable experimental systems and analysis tools to dissect how three-dimensional (3D) tissue environments impact pathogen spread are lacking. Here we develop an Integrative method to Study Pathogen spread by Experiment and Computation within Tissue-like 3D cultures (INSPECT-3D), combining quantification of pathogen replication with imaging to study single-cell and cell population dynamics. We apply INSPECT-3D to analyze HIV-1 spread between primary human CD4 T-lymphocytes using collagen as tissue-like 3D-scaffold. Measurements of virus replication, infectivity, diffusion, cellular motility and interactions are combined by mathematical analyses into an integrated spatial infection model to estimate parameters governing HIV-1 spread. This reveals that environmental restrictions limit infection by cell-free virions but promote cell-associated HIV-1 transmission. Experimental validation identifies cell motility and density as essential determinants of efficacy and mode of HIV-1 spread in 3D. INSPECT-3D represents an adaptable method for quantitative time-resolved analyses of 3D pathogen spread.

## Abstract (web summary)

Here, using an integrative experimental and computational approach, Imle et al. show how cell motility and density affect HIV cell-associated transmission in a three-dimensional tissue-like culture system of CD4^+^ T cells and collagen, and how different collagen matrices restrict infection by cell-free virions.

## Introduction

In molecular biology, reconstituting complex machinery by assembling its different components to a functional unit in vitro serves as ultimate proof for the achievement of overarching understanding. Intense efforts in all the life sciences aim at gaining similarly comprehensive insight into complex physiological processes involving large numbers of heterogenous cells and tissues. Building on knowledge derived from in vivo analyses, one approach is to use ex vivo tissue explants as physiological surrogates^1^. Since organotypic cultures often remain largely refractory to experimental manipulation of tissue organization and composition, parallel and complementary efforts aim at reconstituting complex physiology by assembly of components ex vivo^2^. These novel culture systems allow studying physiological processes at the molecular, single-cell and cell population level. Dissecting the contribution of individual processes on different complexity levels to the overall dynamics requires a systems level understanding of the factors involved. Mathematical and computational models have proven indispensable for connecting experimental data that are generally limited in spatial and/or temporal resolution in order to reveal and quantify physiological dynamics within multicellular systems. These approaches are intensively developed and applied in, e.g., cancer and immunology research^3,4^, but have been much less adopted by other areas such as infectious disease research.

Pathogen spread in the infected host is a particular complex example for multifaceted interactions that can, depending on the balance between key parameters, result in disease or asymptomatic host control. Particularly for obligate intracellular pathogens such as viruses, but also cell-associated stages of many bacteria and parasites, intrinsic host cell behavior and local tissue environment likely present important determinants for the efficacy of pathogen spread. However, little information is available on such environment–pathogen interactions. In the case of human immunodeficiency virus type 1 (HIV-1), many molecular aspects governing replication in isolated target cells, as well as innate and adaptive immune mechanisms of the host and viral evasion mechanisms have been elucidated^5^. In contrast, little is known about (i) the impact the 3D environment exerts on these processes in different physiological target tissues and (ii) whether and how the virus adapts its replication and transmission strategies to such varying environments^6^. HIV-1 spread was initially thought to occur mostly via cell-free virus particles. More recently, it was revealed in in vitro cell culture models that cell-associated transmission modes via physical contact between infected donor and uninfected target cells are more efficient^7–11^. For cell-associated transmission, donor and target cells typically engage in a close cell–cell contact referred to as virological synapses (VS) for the polarized release of virus particles^12^. How tissue environments impact on virus spread and the modes of viral transmission remains to be established.

These gaps in our understanding of fundamental principles governing HIV-1 spread in the host reflect the difficulty to dissect physiological mechanisms in currently available experimental systems or from clinical data. Studies in HIV-1 patient cohorts or experimental infection of continuously improving animal models or target tissue explants allow establishing correlations to characterize important determinants of pathogenesis^6,13^. However, key parameters such as cell density or biophysical properties and composition of target tissue remain largely refractory to experimental control or modification. On the other hand, standard two-dimensional (2D), monotypic cell culture systems offer experimental control of many parameters but lack tissue heterogeneity and organization. For example, such experimental systems neglect that CD4 T cells are highly motile in vivo^14^, and do not allow assessing the impact of HIV-induced reduction of T cell motility on infection spread^15–17^. In addition, gravity rapidly converts standard suspension cultures of CD4 T cells into monolayers with dense cell packing. Within the concept of cell-associated HIV-1 transmission, culturing cells in such high density likely overrides the requirement for motility of donor and target cells to form cell–cell contacts for virus transmission. Importantly, T-cell motility is also the main reason why observations of successful transfer of viral material between motile cells could not be coupled to analyzing whether this transfer leads to productive infection: by the time viral gene expression in the target cell can be detected, donor and target cells have moved away from the disassembled VS, precluding identification of the causative contact^11,17,18^.

**Fig. 1:**
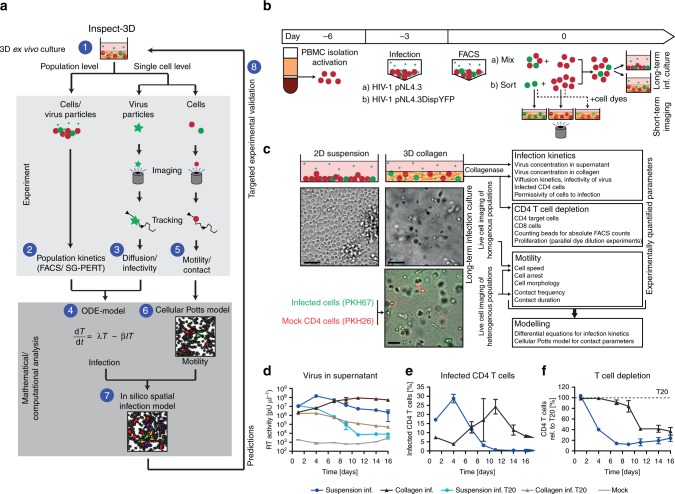
INSPECT-3D workflow, experimental system and population analysis of pathogen spread in 3D collagen. **a** Schematic workflow depicting the different steps of INSPECT-3D. Step 1: Establishment of a 3D culture system (here: primary human CD4 T lymphocytes in collagen to study HIV-1). Steps 2 and 3: Generation of quantitative and kinetic data on pathogen spread on the population level (step 2) and on the level of single pathogens (step 3). Step 4: Use quantitative and time-resolved information from steps 2 and 3 to parametrize a model based on ordinary differential equations (ODE) describing the kinetics of pathogen spread. Step 5: Generation of quantitative and kinetic data on cell motility and contact parameters. Step 6: Use quantitative and time-resolved information from step 5 to generate a cellular Potts model describing cell motility. Step 7: Combine both modeling approaches (steps 4 and 7) into an in silico integrated spatial infection model. Step 8: Experimental validation of predictions from the in silico integrated spatial infection model. Infected cells: green, target cells: red, virus: green asterisks, CD8 cells: purple, newly infected cells in eclipse phase: orange. **b** Schematic overview of experimental procedure of PBMC isolation, activation, infection and set up of long-term infection cultures or short-term imaging. Infected T cells (green) can be separated from uninfected T cells (red) by MACS sorting of cells infected with the HIV-1 variant NL4.3Disp.YFP. **c** Schematic overview of the parameters that can be quantified by INSPECT-3D. Depicted in the top panel are schematic views of 2D suspension and 3D collagen cultures with uninfected and infected cells in red and green, respectively. Asterisks: virus. The middle panel shows a wide-field micrograph of cell density and arrangement, the lower panel a still image from a movie of a 3D culture with uninfected (red, PKH26 stained) and infected (green, PKH67 stained) cells. Scale bars: 40 µm. **d**–**f** HIV-1 spread in suspension or collagen. **d** Virus concentration in supernatants determined by SG-PERT. **e** Percentage of infected (p24+) CD4 T cells determined by flow cytometry. **f** T cell depletion expressed as residual CD4 T cells relative to the respective T20 control, which was set to 100% (dashed line). Mean and standard deviation from parallel triplicate infections of cells from the same donors are shown

To overcome these experimental barriers for studying pathogen spread in physiological conditions we introduce here INSPECT-3D, an Integrative method to Study Pathogen spread by Experiment and Computation in Tissue-like 3D cultures, and apply its stepwise protocol to the analysis of HIV-1 spread (Fig. 1a). We establish 3D collagen matrices as a synthetic and tunable experimental system for analyzing HIV-1 spread ex vivo in primary human CD4 T lymphocytes within a defined 3D environment (step 1). Experimental quantification of critical parameters over 3 weeks characterizes virus spread on the level of the bulk cultures (step 2), as well as individual pathogen (step 3) and cell dynamics (step 5) by single particle and cell tracking. Since the complexity of relevant parameters defining the spread of infection precludes their comprehensive experimental analysis, the experimental data is used to parametrize mathematical models describing population dynamics (step 4) and single-cell motility (step 6). Combining both models into an integrated spatial infection model (step 7) allows predicting previously unrecognized key parameters governing HIV-1 spread, such as cell-contact requirements and target cell motility, which are validated experimentally (step 8). Applying INSPECT-3D to HIV-1 replication in 3D collagen cultures of T lymphocytes reveals that the 3D environment restricts infection with cell-free HIV-1 but promotes the formation of long-lasting cell contacts for transmission of cell-associated HIV-1. In addition, these analyses provide a quantitative, time-resolved understanding of the infection dynamics. The approach is vital for gaining insight into pathogen spread in multicellular systems and is adaptable to a broad range of viral, bacterial, and parasitic pathogens.

## Results

### Ex vivo 3D cultures for HIV-1 spread in primary CD4 T cells

As a first step toward investigating HIV-1 spread in tissue-like environments (step 1, Fig. 1a), we established cultures of human CD4 T lymphocytes in a 3D matrix in which individual experimental parameters can be controlled and compared with standard suspension culture conditions. Collagen was used as 3D matrix because of the following characteristics: Collagen I is the most abundant fibrous protein in healthy interstitial tissue^19^ and can be polymerized into matrices of different densities. Collagen cultures are well-established to study T lymphocyte motility and function in 3D^20,21^ but are not frequently used in infectious disease research. These cultures are amenable to live-cell imaging^15,22,23^ and provide a scaffold for T-cell migration, but—based on the spacing of filaments—should not hinder diffusion of cell-free HIV-1 particles. Moreover, collagen can be dissolved by collagenase to retrieve cells for phenotypic characterization^24,25^.

Human peripheral blood mononuclear cells (PBMCs) from healthy blood donors were activated by a combination of PHA and anti-CD3 antibody (see Methods) to result in ~95% primary human CD3 T cells of which typically 72–76% were CD4 T cells and 18–22% were CD8 T cells. The amount of CD8 T cells present in the culture served as overall indicator of cell health and enabled to monitor CD8 T-cell expansion which is also observed during acute and chronic HIV-1 infection in patients^26,27^. These cultures were infected with either wild-type HIV-1 NL4.3 or with the replication-competent HIV-1 variant NL4.3DispYFP (Supplementary Fig. 1a). The latter encodes for all viral proteins and in addition expresses membrane-associated EYFP at the surface of virus producing cells. Sorting of infected cells via DispYFP using antibody-coupled magnetic beads turned out to be more efficient than other tags and can be achieved without the need for operating specialized equipment such as cell sorters under elevated biosafety conditions (Fig. 1b, Supplementary Fig. 1a, b). First round infected and uninfected cells were mixed in defined amount and ratio, and cultured in suspension or embedded in 3D collagen at a density of 1×10^5^ cells per 100 µl (Fig. 1b). While culturing T cells in suspension leads to high cell density at the bottom of the culture dish, embedding the same number of cells in 3D collagen resulted in well-defined cell spacing. Under these 3D culture conditions, cells remained viable for up to 3 weeks (Supplementary Fig. 1c). Analysis of the cell culture supernatant and matrix allowed for quantification of virus production over time, and cells could be re-isolated from the collagen matrix for phenotypic characterization of cell and virus populations (e.g., determination of relative and absolute numbers of specific cell populations by flow cytometry) (Fig. 1c, see gating strategy in Supplementary Fig. 1d). In addition, the good optical properties of collagen allow for quantification of virus diffusion, cell migration, as well as formation of cell–cell contacts between infected and differentially labeled donor and uninfected target cells by live-cell microscopy. The analysis of pathogen spread in 3D collagen cultures therefore provides quantitative information on the entire cell and pathogen population, but also at the level of single cells and pathogens (Fig. 1a, c).

### 3D culturing affects HIV-1 spread kinetics

Quantification of HIV-1 replication on the level of the bulk culture (step 2, Fig. 1a) indicated robust virus replication and active viral spread characterized by continuous production of virus particles (Fig. 1d). Virus replication was compared with cultures treated with the HIV-1 fusion inhibitor T20 from day 0 to limit virus production to cells that were infected during the initial round of infection. Replication kinetics in collagen were markedly delayed relative to suspension, reached similar maximum titers 1 week later (Fig. 1d, Supplementary Fig. 2a), and showed overall reduced virus replication (Supplementary Fig. 2b). The delayed replication kinetics in 3D collagen was reflected in a delayed increase in infected CD4 T cells (Fig. 1e, Fig. S1 Supplementary Fig. 2c), was associated with a moderate reduction in overall infection in most donors (Supplementary Fig. 2d), and resulted in markedly delayed CD4 T-cell depletion with less pronounced overall loss of CD4 T cells over the course of the experiment (Fig. 1f, Supplementary Fig. 2e). The delay of HIV-1 spread by the 3D environment was not caused by a general impairment of T cell permissivity to HIV-1 infection, as cells cultured in collagen were even slightly more susceptible to HIV-1 infection than cells in parallel suspension cultures (Supplementary Fig. 2f–h). 3D collagen environments thus strongly affect the kinetics of HIV-1 spread.

### HIV-1 particle diffusion and infectivity in 3D collagen

**Fig. 2:**
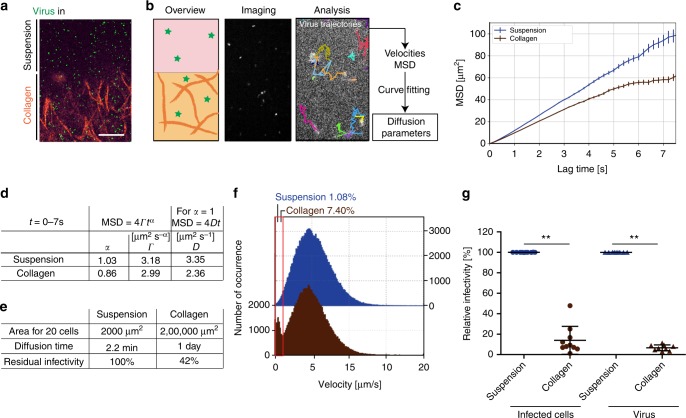
Step 3: 3D collagen limits HIV-1 particle diffusion and infectivity. **a** Representative confocal micrograph of fluorescent viral particles in media or within collagen. Fluorescent viral particles (green, HIV^GFP^ or HIV^mcherry^) were incorporated into collagen (orange) or resuspended in medium overlaying the collagen matrix (suspension). Collagen was visualized using confocal reflection microscopy. Maximum projection of 10 µm stacks at 0.5 µm intervals is shown. Scale 5 µm. **b** Schematic overview of virion diffusion analysis. Motility of viral particles within collagen or in medium (suspension) was monitored by spinning disc confocal microscopy (×40 objective) recording images of one focal plane at maximum speed for 5 min (corresponding to 1475 frames; middle panel). **c** Viral particles were automatically tracked by single particle tracking (see Methods for details). Diffusion parameters were determined from mean square displacement (MSD) computed for at least 20,000 viral tracks (mean+/−SEM). **d** Determined anomalous diffusion exponent α, which is 1 for normal diffusion, scaled transport coefficient Г and diffusion coefficients D. **e** Space and time considerations for virus diffusion in suspension and collagen, taking into account the infectious half-life of HIV-1 of 17.9 h^63^. **f** Distribution of instantaneous velocities of single particles from tracks with durations between 0.8 and 15 s for virus in suspension (blue) or in collagen (brown). Boxed are number of tracking steps with velocities below 1 µm s^−1^. **g** Relative infectivity of HIV-1. Virus that had diffused out of collagen was tested for infectivity normalized for RT activity, using TZM-bl reporter cells in a blue cell assay. Values were normalized for virus amount as assessed by RT activity. HIV-1 was either produced by infected PBMCs cultured in collagen or suspension (infected cells), or sucrose-purified HIV-1 (virus) was directly embedded within media (suspension) or collagen for 24 h. Relative infectivity per µU RT is plotted relative to suspension, which is set to 100%. Mean+/−SD is indicated. Individual symbols indicate independent experiments. Wilcoxon matched pair test was used for statistical analysis

In search for explanations of the delay in HIV-1 spread in 3D collagen we developed approaches to address if 3D collagen affects the availability of cell-free virus particles for infection (step 3, Fig. 1a). Since infection of target cells by cell-free virus requires their diffusion in the culture, we first determined the diffusion properties of fluorescently labeled HIV-1 particles (pcHIV^GFP^ or pcHIV^mcherry^) in medium (suspension) or upon incorporation into 3D collagen (Fig. 2a) by spinning disc confocal microscopy. Images were subjected to automated probabilistic particle tracking, resulting in trajectories of individual HIV-1 particles that were used for mean square displacement (MSD) analysis. Subsequent curve fitting revealed the ability of HIV-1 particles to diffuse in 3D collagen without accumulation at, e.g., collagen fibers and quantified viral diffusion (Fig. 2a, b)^28^. Normal diffusion was observed for virions in suspension (linear MSD curve, diffusion exponent *α* = 1.03) and the obtained diffusion coefficient *D* = 3.18 µm² s^−1^ was comparable to theoretical expectations based on the used media conditions (*D* = 4.38 µm² s^−1^, Stokes–Einstein equation for virions of 150 nm diameter at 37 °C in water) (Fig. 2c, d). We typically started our experiments with 5% (1 out of 20) infected donor cells and hence considered how much time virions spend to reach the 19 nearest neighbors of a virus producing cell. Based on the determined diffusion rate, this would require about 2.2 min in the dense packing of suspension cultures (Fig. 2e). In collagen, however, diffusion occurred at reduced rates (anomalous diffusion exponent *α* = 0.86, Fig. 2c, d) and indicated a pattern of subdiffusion, which is typical for solute transport in porous media^29^. Considering the cell spacing in 3D collagen, diffusion of virions from a producer cell to the 19 nearest neighbors would require almost 1 day (22.6 h). Based on the half-life of HIV-1 particle infectivity of 17.9 h^30^, this would be associated with 58% loss of infectivity (Fig. 2e). In addition, single-particle tracking revealed that within an observation time of 5 min considerably more viral particles underwent transient phases of low mobility in collagen than in suspension (7.4% of all particles in collagen compared with 1.08% in suspension, Fig. 2f). Since we did neither observe long-lasting virion–collagen interactions resulting in decoration of collagen fibers nor aggregation of virions, these phases of low virion mobility likely represent transient contacts with collagen fibers. Considering these frequent immobilization events detected within this short observation window, it can be assumed that within the infectivity half-life of 17.9 h, virtually all particles will encounter collagen fibers. Although such physical contact with collagen fibers did not alter the half-life of RT activity (Supplementary Fig. 3a), collagen severely compromised the infectivity of HIV-1 particles, both for virus produced and virus directly embedded within the matrix (14.0+/−13.6% and 6.7+/−2.9%, respectively, relative to suspension at 100% relative infectivity, Fig. 2g). Finally, virus production as determined by released RT activity was significantly reduced in collagen compared with suspension cultures (41.3+/−18.9% relative to suspension, Supplementary Fig. 3b). Together, these results revealed that a 3D collagen environment reduces virion diffusion rates and restricts particle infectivity as well as production. 3D environments can thus pose a significant barrier to cell-free HIV-1 infection.

### HIV-1 spread in 3D collagen of different densities

**Fig. 3:**
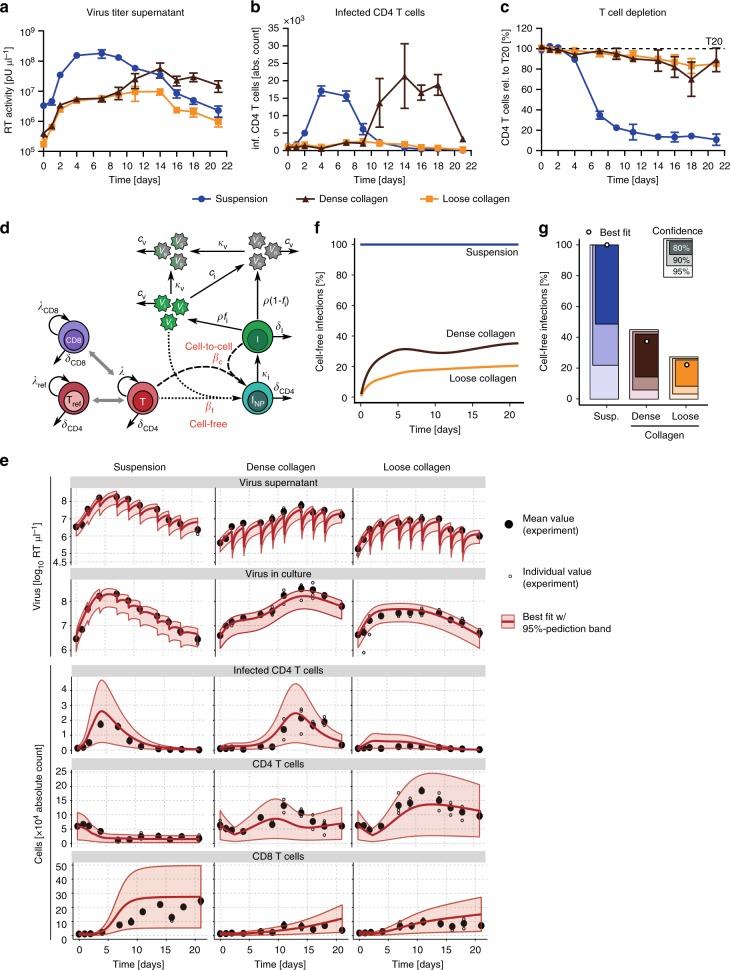
Step 4: Mathematical ODE-model reveals predominant cell–cell spread of HIV-1 in 3D culture. **a**–**c** Cells from a representative donor showing HIV-1 spread in suspension, dense and loose collagen over time. **a** Virus concentrations determined from supernatants by SG-PERT. **b** Absolute numbers of infected CD4 cells as determined by FACS using counting beads. **c** T-cell depletion expressed as residual CD4 T cells relative to the respective T20 control, which was set to 100% (dashed line). Mean and standard deviation from parallel triplicate infections of cells from the same donors are shown. **d** Schematic of the mathematical model describing the infection dynamics within the ex vivo cultures. Target cells (*T*) that proliferate at rate *λ* and die at rate *δ*_CD4_ can become infected (*I*_NP_) by free infectious virus (*V*_i_) at rate *β*_f_ or by contact with infectious cells (*I*) at rate *β*_c_. After remaining in a non-productive infected state for an average duration of 1/*κ*_I_, infected cells start producing virions at a constant rate *ρ* and thereby become infectious. Only a fraction of these particles, *f*_i_, is considered to be infectious, losing infectivity (*V*_n_) at rate *c*_*i*_. Virions are lost from the culture by either disintegrating with a viral clearance rate *c*_V_ or by diffusion to the supernatant (*V*_S_) at rate *κ*_V_. Furthermore, we consider non-permissive refractory CD4 T cells (*T*_ref_) and CD8 T cells that interfere with target cell proliferation (see Methods for a detailed description and the corresponding mathematical equations). **e** Model predictions for the infection dynamics within the different environments. The experimental data indicating the mean (black dots) and individual measurements (open black circles) of three repeats, as well as the best fit (solid red line) of the mathematical model given in (**d**) are shown. Red shaded areas indicate the 95%-prediction bands of model predictions. Note that the repeated drops in virus titer reflect the time points of medium change. **f** Predicted contribution of cell-free infections to total infections for suspension (blue), dense (brown), and loose collagen (orange) over time using the best fit shown in (**e**). **g** Corresponding confidence intervals for estimates of the proportion of cell-free infections three weeks post infection with the prediction of the best fit shown as white circle

To better dissect the impact of different environments on HIV-1 spread, we further exploited the versatility of collagen as matrix for ex vivo culture systems and compared matrices with different collagen concentrations and crosslinking that display high or low confinement (dense and loose collagen), and hence represent a range of heterogeneous tissues. These 3D cultures do not reflect the precise heterogeneity and architecture of a specific target tissue. However, T-cell density and migration speeds are reminiscent of non-lymphoid peripheral, mucosal tissue^31,32^. Surprisingly, we found that loose collagen was more restrictive to HIV-1 spread than dense collagen and allowed only for an initial increase in the production of virus particles that remained at a moderate basal level (Fig. 3a, b, see Supplementary Fig. 4a and 4b for viral replication curves including mock and T20 controls and statistical analysis of results obtained from 10 experiments with individual donors, respectively). This moderate efficacy of virus replication in loose relative to dense collagen was paralleled by low levels of productively infected cells (Fig. 3b) and only subtle depletion of CD4 T cells (Fig. 3c).

### A mathematical model to analyze HIV-1 spread in 3D collagen

The results above revealed that 3D environments shape the efficacy and dynamics of virus spread and individual virion infectivity. However, limitations in either spatial resolution (population-based measurements, step 2, Fig. 1a) or temporal extent (imaging, step 3, Fig. 1a) impair the direct and simultaneous experimental assessment of the relative contribution of individual parameters, such as cell-free and cell-associated viral transmission, to the overall infection dynamics. Overcoming these limitations, mathematical models can provide a systematic and quantitative understanding of the interactive processes within such multicellular systems and represent a tool to quantify parameters that are not directly experimentally accessible (step 4, Fig. 1a). We therefore conducted in-depth mathematical modeling of virus spread kinetics in suspension and 3D collagen to disentangle the effects of replication efficacy, cell motility and contact times on the infection dynamics. Particularly we determined to which extent cell-free and cell-associated viral transmission modes are influenced by the specific environments. Extending previous standard models for viral dynamics (reviewed in ref. ^33^), we developed a kinetic mathematical model based on ordinary differential equations (ODE-model) that describes the dynamics of CD8 cells, uninfected and infected CD4 cells, as well as the concentration of viral particles and their production by infected cells (Fig. 3d). In particular, our mathematical model discriminated infection by cell-free and cell-associated viral transmission^30,34^, distinguished between viral concentrations in matrix and supernatant, and additionally accounted for an “adaptation phase” characterized by delayed proliferation that cells experience after transfer into their environment. Using a stepwise approach by combining our mathematical model with additionally obtained experimental data (see Methods), we were able to reliably quantify the parameters describing cellular turnover and infection kinetics within the different environments (Table 1).

**Table 1:** Estimates for the parameters of the mathematical ODE-model describing HIV-1 infection kinetics within the different environments

Parameter	Description	Unit	Suspension	3D Collagen	
				Loose	Dense
*λ*_CD8_	Proliferation of CD8 cells after adaptation phase	day^−1^	1.13 [0.74, 1.74]	0.58 [0.24, 1.01]	0.29 [0.12, 0.56]
*λ*_CD4_	Proliferation of CD4 cells after adaptation phase	day^−1^	0.60 [0.36, 1.02]	0.78 [0.36, 1.29]	0.59 [0.32, 0.89]
*T*_C_	Max. capacity for CD3 T cells	×10^5^ cells well^−1^	3.11 [2.72, 3.56]	3.53 [2.80, 4.52]	4.28 [3.15, 6.37]
*δ*_CD8_	Net-death rate of CD8 cells during adaptation phase	day^−1^	0.07 [0, 0.19]	0.11 [0, 0.23]	0.08 [0, 0.19]
*δ*_CD4_	Net-death rate of CD4 cells during adaptation phase	day^−1^	0.03 [0, 0.11]	0.23 [0.05, 0.39]	0.25 [0.09, 0.38]
*I*_0_	Initial fraction of non-productively infected cells in T20x2 experiment	1	0.17 [0.10, 0.23]		
*ρ*	Viral production rate	×10^4^ RT (cell × day)^−1^	1.02 [0.80, 1.38]	0.48 [0.37, 0.66]	0.43 [0.32, 0.61]
*δ*_I_	Death rate of infected cells	day^−1^	0.42 [0.40, 0.44]	0.48 [0.46, 0.50]	0.52 [0.51, 0.56]
*κ*_V_	Viral diffusion rate from culture to supernatant	day^−1^	0.72 [0.55, 0.95]	0.13 [0.10, 0.17]	0.15 [0.11, 0.19]
*I*_0_	Initial fraction of non-productively infected cells in WT experiment	×10^−2^	3.3 [1.7, 4.8]		
*V*_0_	Initial fraction of infectious virus in WT experiment	1	4.4 × 10^−8^ [0, *f*_i_ = 10^−3^]		
*T*_ref,0_	Initial fraction of refractory cells in WT experiment	1	0.10 [0.0074, 0.12]		
*β*_f_	Cell-free infection rate	×10^−5^ (RT × day)^−1^	2.3 [0, 3.0]		
*β*_c_	Cell-to-cell infection rate	×10^−5^ (cell × day)^−1^	1 × 10^−6^ [0, 7.0]	4.3 [3.6, 5.4]	1.7 [1.4, 2.6]
*λ*_r_	Proliferation rate of refractory cells	day^−1^	0.22 [0.16, 0.29]	0.78 [0.77, *λ*_CD4_ = 0.78]	0.49 [0.45, 0.52]

The schematic of the model is depicted in Fig. 3d, and the model and fitting procedure is explained in detail in Methods. Numbers in brackets represent 95%-confidence intervals for parameter estimates obtained by profile likelihood analysis

Specifically, our model explains the observed dynamics for all different components measured, including amounts of infected as well as total CD4 T cells, CD8 T cells and virus titers in the cell culture supernatant and the matrix (Fig. 3e, Supplementary Fig. 5a, b). We found that the different environments neither affect the proliferation of CD4 target cells (*λ*^S^_CD4_ = 0.60 [0.36, 1.02] d^−1^ vs. *λ*^L^_CD4_ = 0.78 [0.36, 1.29] d^−1^ vs. *λ*^D^_CD4_ = 0.59 [0.32, 0.89] d^−1^), nor the rate at which infected cells die (*δ*^S^_I_ = 0.42 [0.40, 0.44] d^−1^ vs. *δ*^L^_I_ = 0.48 [0.46, 0.50] d^−1^ vs. *δ*^D^_I_ = 0.52 [0.51, 0.56] d^−1^_,_ numbers in brackets represent 95%-confidence intervals of estimates) (see Table 1). All three environments have a similar maximum capacity for total CD3 T cells (*Τ*^S^_C_ = 3.11 [2.72, 3.56] × 10^5^ cells well^−1^ vs. *Τ*^L^_C_ = 3.53 [2.80, 4.52] × 10^5^ cells well^−1^ vs. *Τ*^D^_C_ = 4.28 [3.15, 6.37] × 10^5^ cells well^−1^). Thus, the high depletion of CD4 T cells in suspension cultures is compensated by a higher proliferation rate of CD8 cells in this environment (*λ*^S^_CD8_ = 1.13 [0.74, 1.74] d^−1^ vs. *λ*^L^_CD8_ = 0.58 [0.24, 1.01] d^−1^ vs. *λ*^D^_CD8_ = 0.29 [0.12, 0.56] d^−1^). Importantly, our model robustly inferred the contribution of cell-free and cell-associated infections to viral spread in suspension and 3D cultures (Fig. 3f, g). In suspension, virus spread was found to be mainly driven by cell-free infection, since no contribution of cell-associated infection was required to explain the experimental data (Fig. 3f, g). In contrast, HIV-1 spread in collagen relied to a large extent on cell-associated modes of virus transmission with a maximum of only 22% [possible range between 0.0% and 27.0%] and 37% [possible range from 0.0% to 44.2%] of infections resulting from cell-free transmission in loose and dense collagen, respectively (numbers in brackets define the 95%-confidence intervals of estimates). The probability for infection of cells by cell-associated transmission is estimated to be more than ~7- and ~4-fold higher in loose and dense collagen, respectively, compared with suspension (Supplementary Fig. 5c). Our kinetic model thus indicated that 3D environments exert strong selection pressures toward the use of cell-associated modes of HIV-1 transmission.

### Single-cell motility and contact analysis

**Fig. 4:**
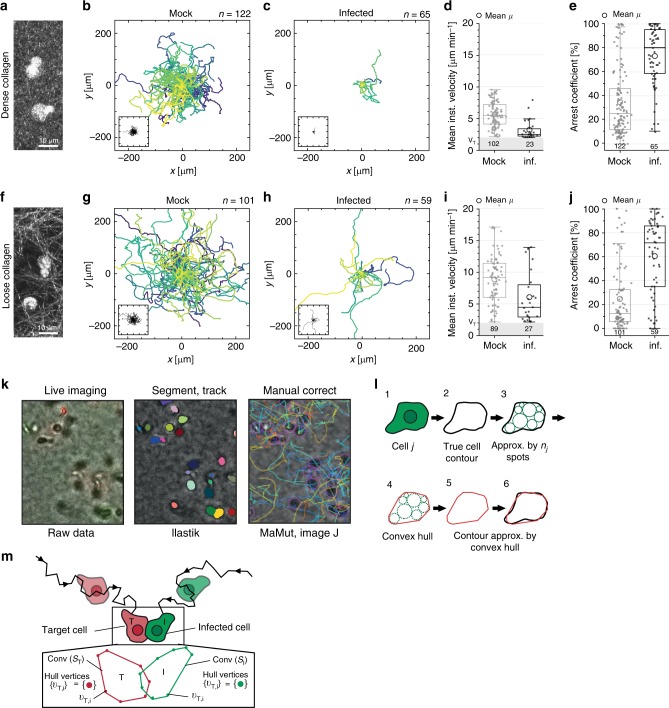
Step 5: Motility analysis in different collagen densities. Cell migration analysis. Mock CD4 T cells and purified HIV-1 infected cells were stained with red and green PKH cell dyes, respectively, mixed and embedded in loose or dense collagen. Cells were imaged every 30 s, segmented and tracked for 60 min. **a**, **f** Maximum projection of 10 µm stacks of confocal reflection microscopy showing lymphocytes in a compact collagen meshwork of dense collagen (**a**) and in a fibrillary structure in loose collagen (**f**). Scale bar 10 µm. **b**, **c**, **g**, **h** Trackplots of mock infected and infected CD4 cells in dense and loose collagen. The inset shows a zoom-out to visualize the maximal extent of the longest tracks. The analyzed cell number is indicated by *n*. **d**, **i** Mean instantaneous velocity of motile cells (faster than 2 µm min^−1^ as minimum velocity marked by gray bar). **e**, **j** Arrest coefficient of all cells indicating the fraction of track lengths displaying arrest (<2 µm min^−1^). Absolute numbers of cells analyzed are indicated below the boxes. Box and whiskers in Tukey style with mean values indicated by circles are shown. **k** Workflow for advanced segmentation and tracking. From left to right: Wide-field image from live microscopy, overlay with segmentation result as computed using Ilastik, manual track correction with Image J plugin MaMut. See Methods for details. **l** In case one cell was detected by multiple spots, these were joined to compute a convex hull used for contact analysis. See Methods for details. **m** Definition of contact based on overlapping cell shapes as approximated by convex hulls

The kinetic mathematical model indicated a comparable or even slightly higher contribution of cell–cell transmission in loose than in dense collagen cultures and cell-free infectivity was equally impaired by dense and loose collagen. The slower virus spread observed in loose collagen therefore suggested that cell–cell transmission is particularly inefficient under this culture condition. Due to the spacing between donor and target cells in 3D, cell motility is a prerequisite for cell-associated virus spread in these 3D environments. Understanding the kinetics of virus spread in 3D hence required analyzing cell motility and cell–cell interactions at the single-cell level. We therefore employed live-cell wide-field microscopy to analyze the motility of infected and uninfected cells in 3D (step 5, Fig. 1a). In dense collagen (Fig. 4a), elevated constriction confined cell motility that resulted in frequent turning and moderate cell speeds (Fig. 4b–d). Consistent with previous observations in vitro and in vivo^15–17,35^, HIV-1 infection impaired T-cell migration leading to reduced cell velocities (mock: 5.6+/−2.0 µm min^−1^, HIV-1: 43.2% reduction in cell speed to 3.2+/−1.6 µm min^−1^, Fig. 4d, Supplementary Movie 1) and longer arrest phases (Fig. 4e). In comparison, lower constriction in loose collagen (Fig. 4f) allowed for overall enhanced cell migration, resulting in more even and extended migration tracks (Fig. 4g, h). Overall migration was also faster (mock: 8.9+/−3.8 µm min^−1^, HIV-1: 31.6% reduction to 6.1+/−4.07 µm min^−1^, Fig. 4i), since cells underwent less arrest phases than in dense collagen (Fig. 4j, compare 24.7+/−28.4% in loose to 34.9+/−28.5% for mock cells in dense collagen, Fig. 4e, Supplementary Movie 2). With a 2.1- and 2.5-fold increase in arrest coefficient for dense and loose collagen, respectively, the reduction of T-cell motility by HIV-1 infection was comparable for both collagen densities (Fig. 4e, j). However, less constriction in loose collagen resulted in overall higher migration efficiencies for both infected and uninfected cells.

**Fig. 5:**
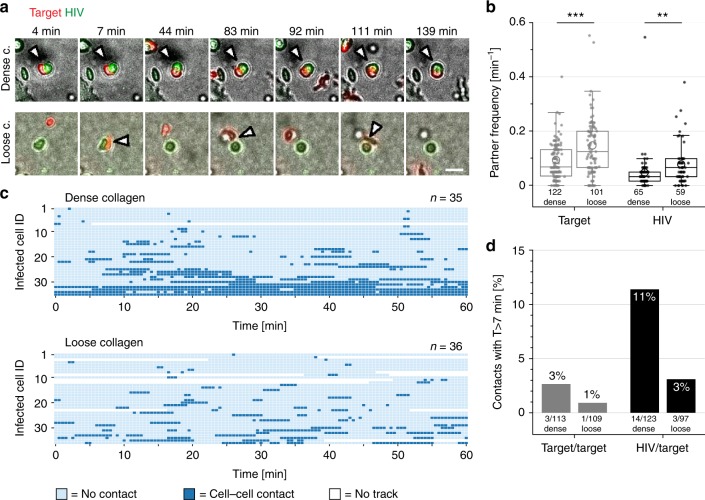
Step 5: Cell contact analysis in different collagen densities. Contact analysis of target CD4 T cells in co-culture with HIV-1 infected donor cells. Mock CD4 T cells and purified HIV-1 infected cells were stained with red and green PKH cell dyes, respectively, mixed and embedded in loose or dense collagen. Cells were imaged every 30 s, segmented and tracked for 60 min. **a** Still images of supplementary movie 3 recorded at the indicated times. Green: HIV-1 infected cell (HIV), red: target CD4 T cell (target). Contacts are indicated by an arrowhead. Scale bar 20 µm. **b** Partner frequency per min of target or donor cells with other cells in dense and loose collagen. Numbers indicate absolute cell number. The graph shows boxplots with Tukey style whiskers, circles indicate mean values. Mann–Whitney test was used for statistical analysis. **c** Contact diagram for donor cells with target cells in dense and loose collagen. Individual cell tracks are color coded: light blue, without contact; dark blue, in contact with another cell; white, the track has ended/not yet begun. **d** Frequency of contacts lasting longer than 7 min between two uninfected cells (target/target) or infected/uninfected cell pairs (HIV/target). Numbers indicate absolute cell contact number

As cell-associated transmission was identified to dominate HIV-1 spread, live-cell imaging was used to investigate cell–cell contact formation. Green and red cell dyes were used to label HIV-1 infected and uninfected T cells, respectively, and to monitor cell contacts between both cell types after mixing. Analyzing contact parameters in a quantitative manner required the implementation of customized advanced segmentation and tracking algorithms in Ilastik with subsequent manual track correction with the Image J plugin MaMut (Fig. 4k). Cell tracking resulted in cell trajectories with potentially multiple circular spots per cell and per time point. The true cell contour was then approximated by the convex hull of all spots of a given cell (Fig. 4l), such that the cell shape could be followed over time. Cell–cell contacts were then detected as overlapping cells, which were identified by mutually overlapping convex hulls (exemplary illustrated in Fig. 4m for a red target cell in contact with a green infected cell. See Methods for details on the contact analysis). As expected, faster migration in loose collagen resulted in a higher frequency of cell–cell contacts for both uninfected target (target) and infected donor (donor) cells than observed in dense collagen (Fig. 5a–c). However, cell contacts between donor and target cells in dense collagen occurred more frequently within very compact and stable aggregates, while contacts were more dynamic and short-lived in loose collagen (Fig. 5a, c, d, Supplementary Movie 3). Formation of stable cell–cell contacts (arbitrarily defined as lasting at least 7 min) was approximately three times more frequent between donor and target cells than between two target cells in both collagen environments (Fig. 5d). Importantly, such stable donor-target cell contacts were over three times more frequent in dense than in loose collagen (Fig. 5d) and donor-target cell contacts lasting for at least 10 min were observed exclusively in dense collagen (Supplementary Fig. 6a). For contacts between two target cells, the cumulative cell-contact duration was similar between loose and dense collagen with only a minority of cells engaged in cell–cell contacts for more than 20% of the observation period (Supplementary Fig. 6b). Shorter contact duration was therefore compensated by a higher partner frequency in loose collagen. While a similar pattern was observed for donor-target cell contacts in loose collagen, >30% of donor cells were in contact with target cells for more than 30% of the observation period (Supplementary Fig. 6c). This resulted in a 2.4-fold higher cumulative contact duration between donor and target cells in dense collagen than in loose collagen (Supplementary Fig. 6d). Live-cell imaging and individual cell tracking thus revealed that fast migration in loose collagen is associated with short contact duration and that more efficient virus spread in dense collagen is associated with extended contact duration between donor and target cells.

### An in silico integrated spatial model for HIV-1 spread

**Fig. 6:**
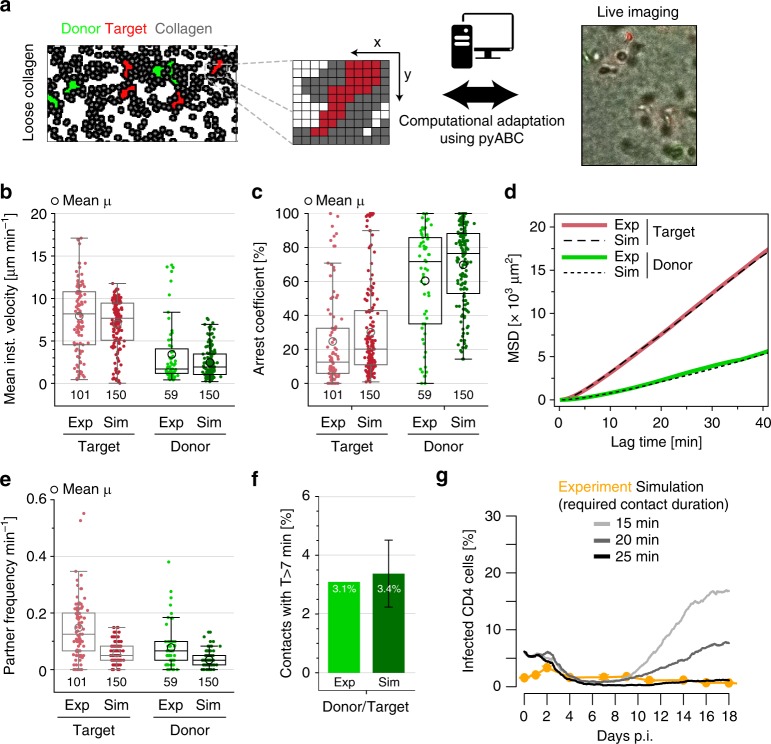
Steps 6 and 7: CPM recapitulates cell migration in loose collagen and in silico spatial infection model determines minimal contact duration for productive cell-to-cell spread. **a** Snapshot of the CPM, which is a lattice-based model with individual cells consisting of several grid sites. Shown are loose collagen conditions with infected donor cells (green), target cells (red), and collagen (gray). The CPM is automatically adapted to live-cell imaging data using the pyABC-framework. **b**–**d** Motility characteristics showing the mean instantaneous velocity (**b**), arrest coefficient (**c**), and mean square displacement (**d**) of target and donor cells comparing experimental data and simulated cells. Simulated data comprise the results of one representative simulation starting with 150 donor and target cells each and followed over 1 h based on the parameterization obtained by fitting the CPM to the experimental data. **e** Simulated cell tracks show comparable partner frequencies between target and donor cells. **f** Proportion of long-lasting cell contacts (>7 min) for donor-target contacts. **g** Simulated long-term infection dynamics using different assumptions for the minimal contact duration between infected and uninfected cells allowing cell-to-cell spread of infection. For each contact duration, the mean over 10 independent simulation runs is shown. Experimental data are depicted in yellow. Boxplots show Tukey style whiskers, circles indicate mean values

Determining how single-cell motilities and contact kinetics obtained from time-restricted live-cell imaging relate to overall population dynamics requires an integrative method that is able to examine multicellular dynamics over long time periods. Computational models that follow the collective dynamics of individual cells provide such a framework. We developed a cellular Potts model (CPM) that provides a computational simulation of cell motion accounting for biophysical properties of individual cell types^3,36^ (step 6, Fig. 1a). In contrast to the kinetic ODE-model developed above, the CPM allows us to directly simulate cell-associated virus transmission between cells migrating in collagen matrices by reflecting the spatial details of individual cell migration and contact dynamics. As loose collagen conditions were most restrictive to infection, we recapitulated these conditions by using an automated parallelized computing method to adapt the CPM to the experimentally observed motility characteristics of infected and uninfected cells obtained from time-lapse imaging (Fig. 6a, Supplementary Movie 4). The obtained parameterization closely mimicked important parameters of motility of individual cells, such as cell velocity, arrest and MSD (Fig. 6b–d). Moreover, corroborating the appropriateness of the obtained parameterization, the CPM adjustment resulted in simulated rates of partner encounters for uninfected and infected cells (Fig. 6e) and percentages of long-lasting contacts between infected and uninfected cells (3.4% (sim) vs. 3.1% (exp) of all contacts) (Fig. 6f) that were in good agreement with our experimental data. To investigate the influence of various cell-contact requirements on the long-term infection dynamics, the CPM adjusted to the individual cell motility characteristics could now be extended to recapitulate the experimental infection assays. To this end, we incorporated a cell–cell infection process into our CPM and accounted for the particular processes and rates deduced by our kinetic ODE-modeling approach (Table 1). These parameters included an estimated viral eclipse phase in which cell-associated transfer from infected to target cells does not yet occur and cell proliferation rates, as well as a maximal cell density that can be reached in the culture system (culture capacity) (see Methods for the specific details of recapitulating experimental conditions). Following this approach allowed us to assemble dynamic and time-resolved information from population-based and single-cell measurements in an integrative spatial infection model to dissect the dynamics of pathogen spread (step 7, Fig. 1a). Within this model we could vary the minimal contact duration required for productive cell–cell virus transmission from donor to target cell and extrapolate infection kinetics. These analyses predicted that loose collagen conditions would be well permissive to HIV-1 spread if productive infection would only require 15 min. This suggested that (i) longer lasting cell contacts are required for productive HIV transmission and thus (ii) only a fraction of contacts defined above as long-lasting (more than 7 min) lead to productive infection of target cells. Expanding the minimal contact duration required for productive HIV transmission to 25 min approximated experimental infection kinetics (Fig. 6g). Thus, our computational procedures identified ~25 min as the lower threshold for the duration of cell–cell contacts leading to productive HIV-1 transmission in 3D collagen.

### Experimental validation of the in silico infection model

**Fig. 7:**
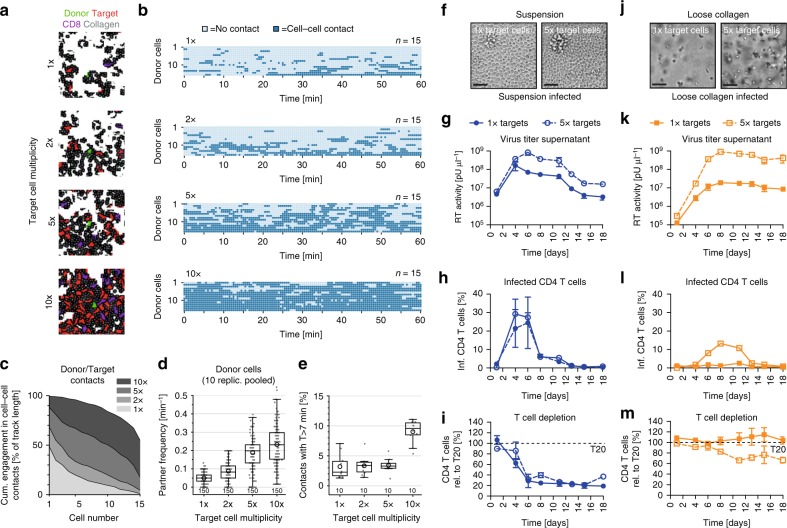
Step 8: Experimental validation of model prediction shows dependency of HIV spread on target cell concentrations in loose collagen. **a** Snapshots of in silico spatial infection model simulations for loose collagen (gray) using a constant number of infected donor cells (green) mixed with varying concentrations (1×–10×) of uninfected CD4 target cells (red) and CD8 cells (purple). **b** Contact diagram for simulated donor cells with target cells in loose collagen for varying target cell concentrations. Individual cell tracks are color coded: light blue, without contact, dark blue, in contact with another cell. One representative simulation of 10 replicates is shown. **c** Corresponding cumulative cell–cell contact durations of simulated donor cells from panel (**b**). **d** Partner frequency of individual HIV-1 infected donor cells pooled from 10 independent simulations with 15 cells each (150 cells total) followed over 1 h for each concentration of target cells. The graph shows boxplots with Tukey style whiskers, circles indicate mean values. **e** Proportion of long-lasting cell contacts (>7 min) between infected and target cells from 10 independent simulations. **f**–**m** Experimental data for comparison of the infection dynamics in suspension (blue, **f**–**i**) and loose collagen (orange, **j**–**m**) using the standard (1×, solid lines) and fivefold increased (dashed lines) concentration of target cells. Scale bar of representative bright-field images: 40 µm. **g**, **k** Virus concentrations determined from supernatants by SG-PERT. **h**, **l** Percentage of infected (p24+) CD4 T cells determined by flow cytometry. **i**, **m** T cell depletion as residual CD4 T cells relative to respective T20 control, which was set to 100% (black dashed line). Mean and standard deviation from parallel triplicate infections of cells from the same donors are shown

If faithfully reflecting the dynamic processes governing pathogen spread in 3D, the integrative spatial infection model should predict important parameters that govern the spread of infection (step 8, Fig. 1a). To test this, we sought to dissect the relevance of cell density on virus spread and simulated cell–cell interactions using a constant amount of infected cells mixed with a 2-, 5- and 10-fold increased number of uninfected target cells (Fig. 7a). As expected, increased target cell densities were generally predicted to expand the time that infected donor cells spend in contact with uninfected target cells, as contacts become more frequent (Fig. 7b–d). A two-fold higher target cell concentration only led to moderate changes in the contact characteristics. In contrast, a five-fold higher number of target cells substantially increased the cumulative time an infected cell spent in contact (average cumulative contact time per infected cell per hour: 16.3 min or 27% of the time (1×); 21.4 min/36% (2×); 38.3 min/64% (5×)) (Fig. 7c) as well as the frequency of different partners that are detected during this time period (Fig. 7b, d). Although the fraction of long-lasting contacts between infected and uninfected cells was predicted to be unaltered by this fivefold increase in target cell density (Fig. 7e), simulating the long-term infection dynamics for different multiplicities of target cells suggested that under these conditions, HIV-1 spreads efficiently in loose collagen (Supplementary Fig. 7a, b). Substantially higher target cell concentrations (10×) leading to densely packed environments even increased this efficiency by additionally affecting cell contact characteristics (Fig. 7d, e). These results suggested that limited average durations of individual donor-target cell contacts can be compensated by an enhanced frequency of cell–cell contacts. To test this assumption experimentally, we compared the infection dynamics within suspension (Fig. 7f–i) and loose collagen (Fig. 7j–m) using the standard and fivefold higher concentration of target cells (data with T20 and mock controls plotted to compare suspension vs. collagen at the same target cell multiplicity in Supplementary Fig. 7c–j). In suspension, virus production over time (Fig. 7g), the percentage of infected cells (Fig. 7h) and CD4 T-cell depletion (Fig. 7i) followed the same dynamics for both cell densities, indicating that the target cell concentration does not affect efficient viral spread in suspension. In contrast, enhancing target cell density in loose collagen, and thus creating an environment that is reminiscent of local T cell accumulation at foci of SIV infection within the macaque vaginal mucosa^37^, increased the abundance of infected cells (Fig. 7l). As predicted by the CPM, this increase in target cell density induced efficient virus spread that was significantly enhanced compared with the basal replication observed at lower cell density as assessed by virus production (Fig. 7k) and CD4 T-cell depletion (Fig. 7m). This experimental validation of the integrative spatial infection model underscores that by applying the full INSPECT-3D analysis, quantitative and time-resolved understanding was achieved of key parameters that govern HIV-1 spread in tissue-like microenvironments. Together, these results establish environmental impact on cell-associated modes of HIV-1 transmission and cell density as central determinants of virus spread in 3D cultures.

## Discussion

Studying pathogen spread in complex cell systems to understand the factors determining the observed dynamics requires an integrative approach combining experimental observations from different complexities and resolutions with imaging and computational analyses. Here we introduce INSPECT-3D as a method for the quantitative analysis and mechanistic dissection of pathogen spread in tissue-like 3D cultures. As a first element, 3D cultures of primary human CD4 T cells are introduced as synthetic ex vivo model system to quantitatively study pathogen spread in well-defined and tunable microenvironments of varying characteristics. This culture system provides experimental control over a wide range of parameters including cell vs. matrix density and cell migration speeds. Furthermore, it allows quantitative and time-resolved analysis of pathogen diffusion, infectivity and replication dynamics, live-cell imaging of cell motility and cell–cell interactions, as well as phenotypic characterization of donor and target cells. INSPECT-3D thus yields quantitative and time-resolved information on single cell/pathogen as well as the population level. To facilitate the analysis of these complex imaging datasets, advanced image segmentation and tracking represents a second essential element of INSPECT-3D. Finally, mathematical modeling is fundamental to process these complex experimental datasets to quantify parameters that are not experimentally accessible and to disentangle the influence of many independent processes on the complex dynamics of pathogen spread. These mathematical analyses are required to connect the individual observations from different spatial and temporal resolution within an integrative framework to get a systematic and quantitative understanding of the processes involved in pathogen spread. As a third element of INSPECT-3D, we therefore performed iterative cycles of mathematical analyses and experiments to identify and quantify key parameters governing virus spread efficacy (Fig. 1a). Combined analyses of live-cell imaging data and measurements of long-term infection dynamics with mathematical models allowed addressing the infection dynamics at a cell population level. In combination with the spatially-resolved description of individual cell dynamics, we were able to generate an integrated in silico spatial HIV-1 infection model, which allowed assessing the impact of motility and interaction patterns of individual cells on virus spread.

Herein, we customized INSPECT-3D to the analysis of HIV-1 spread in 3D cultures of primary human T lymphocytes, but this approach will be a rich resource for the entire infectious disease community. Considering the profound impact of the 3D environment on virus spread, culturing T lymphocytes in 3D collagen should be considered as a novel and easy to implement standard culture system for ex vivo replication studies of HIV-1 but also any other lymphocyte-tropic pathogen. The experimental part of INSPECT-3D can readily be applied to other important T-cell tropic pathogens including viruses (e.g., measles virus^38^, human T-cell leukemia virus^39^, dengue virus^40^, and vaccinia virus^41^) or parasites (e.g., Toxoplasma gondii)^42^. Adaptation to B cell-tropic pathogens such as murine leukemia virus^43^ or Epstein-Barr virus^44^, including antigen-presenting cell such as dendritic cells or macrophages to allow studying, e.g., intracellular bacteria (e.g., Listeria, Salmonella^45^), or studies of co-infections with multiple lymphotropic pathogens only requires minor modifications in the cell purification protocol. Since penetration through the dense collagenous tumor interstitium has been found limiting for therapeutic success of oncolytic adenoviruses^46^ and cancer cells have more plasticity in 3D than in 2D, INSPECT-3D can also be applied to optimize such intervention strategies. For these applications, the mathematical models developed here need to be adapted to the pathogen-specific replication and transmission characteristics.

We aimed at establishing and quantitatively characterizing a 3D culture system amenable to experimental variation of key parameters for studying HIV-1 spread in a tissue-like environment. As in any reconstitution attempt of complex biological processes, this prototype model system does not recapitulate all aspects relevant to HIV-1 spread in an infected individual as it lacks, e.g., adaptive and cellular immune responses with their corresponding cytokine milieus, does not reflect the precise tissue architecture and cell heterogeneity/density characteristic for individual target organs, and relies on non-physiological activation stimuli to render CD4 T cells permissive to HIV-1 infection. In addition to expanding the pathogen portfolio subjected to INSPECT-3D analysis, future efforts will therefore focus on sequentially incorporating more aspects of in vivo physiology (e.g., antigen-presenting cells to deliver virus concomitant with antigen-mediated activation of CD4 T cells) in this ex vivo 3D culture model. The precise steps required to enhance the complexity of this 3D ex vivo model will depend on the nature of the target tissue to be simulated, warranting independent efforts to develop surrogates, e.g., mucosal or lymphoid tissue. These developments will be paralleled by enhanced imaging approaches to track cell motility and interaction in 3D as well as corresponding computational efforts to model these more complex scenarios to faithfully reconstitute pathogen spread in distinct target organs ex vivo and in silico.

Despite these differences to in vivo physiology, this new ex vivo 3D culture system recapitulated the motility speeds of CD4 T lymphocytes and their reduction upon HIV-1 infection observed in humanized mice^17^. This allowed us for the first time to take into account the impact of cell motility on the efficacy of HIV-1 spread and provided important new insights into the mechanisms that govern HIV-1 spread in a 3D environment that are likely important for virus spread in vivo. Most importantly, our analysis revealed a potent influence of the 3D environment itself as it suppressed the infectivity of cell-free HIV-1 particles and posed significant barriers to virion diffusion as well as cell motility. The current view on barriers to HIV-1 spread in infected individuals is primarily focused on cellular, adaptive, and cell-intrinsic mechanisms^5,47^. Our results suggest that extracellular microenvironments can pose similar restrictions to virus spread. This interference does not require pathogen recognition or adaptive immune responses but is an intrinsic physical property of the extracellular matrix, for which we propose the term environmental restriction. Since cell-free infection is markedly impaired by the environmental restriction in 3D cultures, HIV-1 spread depends to much larger extend on cell-associated transmission. The latter transmission mode is promoted by dense 3D environments that induce long-lasting cell–cell contacts and possibly select for virus variants with efficient cell–cell transmission properties. While the molecular mechanisms underlying the restriction to cell-free and promotion of cell-associated infection remain to be established, our results suggest that infection by cell-free HIV-1 particles is only a minor contributor to virus dissemination in the infected host and imply that cell-associated modes of HIV-1 transmission largely predominate in tissue. The predominant use of cell-associated transmission would explain how blocking the exit of CD4 T cells from lymph nodes interferes with HIV-1 spread^17^ and the micro-anatomical clustering of virus variants in humanized mice^18^. Finally, these analyses also revealed two novel key characteristics of HIV-1 cell–cell transmission in 3D: (i) individual cell–cell contacts need to last at least 25 min for productive transfer of HIV-1 to target cells and (ii) increasing the target cell density is sufficient to drive HIV-1 replication in a 3D environment in which elevated cell motility precludes formation of a sufficient number of long-lasting cell–cell contacts. Live cell imaging revealed transfer of HIV-1 proteins from donor to target cells for an average time of ~60 min, including shorter transfer periods of 20 min^11,18,48^, but could not define whether such transfer events result in productive target cell infection. Our results reveal such relative short cell–cell contacts to be indeed sufficient for productive virus transmission. This has important implications for the conditions at which HIV-1 can efficiently spread in tissue: While 3D environments facilitate cell-associated virion transfer by restricting cell-free infection, environment-determined parameters can also have negative effects on HIV-1 cell–cell transmission. Elevated cell migration speeds such as in loose relative to dense collagen may reduce the efficacy of cell-associated HIV-1 transmission, possibly reflecting that arrest for VS formation^43^ is more difficult for highly motile cells. Of note, increasing target cell density restored HIV-1 spread in loose collagen by increasing the total number of permissive cell–cell contacts, thus generating a critical population size of infected cells sufficient for infection spread. This requirement for optimized matrix and target cell density for HIV-1 spread in tissue is consistent with the elevated T cell density in SIV infected foci observed in vivo^37^. In addition, the lack of sufficiently long permissive contacts could be compensated for by a series of shorter cell–cell contacts. This scenario resembles the mechanism of triggering T-cell receptor signaling at the immunological synapse, which occurs in the context of a long-lasting, stable synapses formed by stationary cells or via repeated short interactions of motile cells (kinapse)^49,50^. In analogy to the immunological kinapse, sequential interactions between the same target and one or more donor cells (virological kinapse) may result in expanded cumulative contact duration required for productive HIV-1 transmission. The benefit of such virological kinapses may be the sequential accumulation of sufficient virus material without requiring full motility arrest. In addition, initial waves of transmitted virions might saturate target cell restriction factors to facilitate infection by particles transmitted via subsequent contacts.

This proof-of-concept study illustrates the usefulness of engineered 3D environments coupled to mathematical modeling to further our understanding of pathogen spread dynamics in the infected host and to enable the dissection of underlying mechanisms. Applied to HIV-1 spread in CD4 T cells, their use revealed (i) the motility of donor and target cells as important parameters governing the efficacy of HIV-1 spread in 3D, (ii) tissue-like microenvironments as important regulator of mode and efficacy of HIV-1 spread, and (iii) allowed to gain a quantitative and spatio-temporal understanding of HIV-1 spread dynamics in 3D. INSPECT-3D provides the infectious disease community with a framework to exploit the full potential of ex vivo tissue engineering for the analysis of pathogen spread.

## Methods

### Cell lines

HEK293T cells (ATCC, CRL-3216) and TZM-bl indicator cells (NIH AIDS repository^51^, Cat# 8129; RRID: CVCL_B478) were maintained at 37 °C with 5% CO_2_ in DMEM medium supplemented with 10% fetal calf serum (FCS) and 1% penicillin/streptomycin (Gibco). OKT3 hybridoma cells (ATCC, CRL-8001) were maintained in IMDM (ATCC) supplemented with 20% heat-inactivated FCS and 1% penicillin/streptomycin. Cells were passaged every 2–3 days at 70% confluency.

### Primary human T cell isolation and culture

Human peripheral blood mononuclear cells (PBMC) were isolated from buffy coats from healthy individuals, as anonymously provided by the Heidelberg University Hospital Blood Bank in accordance with regulations of the ethics committee of the Medical Faculty of Heidelberg University. Cells were purified by Ficoll gradient centrifugation and washed in PBS. For activation, PBMC were split into three fractions that were individually stimulated with 0.5 µg ml^−1^ PHA, 5 µg ml^−1^ PHA and surface-immobilized anti-CD3 mAb (OKT3 hybridoma supernatant) for 72 h at 3 × 10^6^ cells per ml in RPMI 1640 supplemented with 10% FCS and 1% penicillin/streptomycin (Gibco), all in the presence of 10 U ml^−1^ IL-2. Subsequently, cells were pooled in high-IL-2 medium (10 ng ml^−1^) and subjected to spin-infection. This procedure resulted in ~95% of CD3 T lymphocytes with typically 70–80% CD4 T cells and 20–30% CD8 T cells. For infection with sortable HIV-1 NL4.3.Disp.YFP, three donors were pooled and depleted of CD8 cells according to manufacturer’s protocol (Miltenyi Biotec) prior to activation.

### Viruses

Virus stocks of replication-competent HIV-1 strains (pNL4.3 SF2 Nef, pNL4.3.Disp.YFP) were prepared from transient transfection of 25 µg plasmid DNA into HEK239T cells (subconfluent 15 cm dish) with JetPei (50 µl, Peqlab) or linear polyethyleneimine (PEI, 142 µl of 1 mg ml^−1^, Sigma Aldrich). For single-round HIV-1 (pNL4.3 Δenv VSV-G), 22 µg of pNL4.3 Δenv and 3 µg of VSV-G were used. Two to three days after transfection, supernatants were harvested, filtered (0.45 µm), concentrated via ultracentrifugation through a 20% (w/w) sucrose cushion, gently suspended in PBS, and stored in aliquots at −80 °C. All virus preparations were handled in a Biosafety Level 3 (BSL-3) containment laboratory in accordance with proper BSL-3 safety procedures.

### Virus cloning

The proviral plasmid coding for sortable HIV-1, pNL4.3IRES.Display.YFP (pNL4.3Disp.YFP), is based on pNL4.3IRES.eGFP^52^. Briefly, eGFP was replaced with Display.YFP based on pDisplay Vector (Catalog No. V660-20, Invitrogen) using NcoI and XmaI. While the sortable tag (Display.YFP) allows isolation of infected cells (see below), it is lost after multiple rounds of infection.

### Virus titer quantification by SG-PERT

One step PCR-enhanced reverse transcriptase assay (SG-PERT) was used as a sensitive quantification tool for HIV virus titers and was performed as previously described^53^.

Briefly, concentrated virus stocks were first diluted 1:100 in PBS, whereas culture supernatants were directly lysed in 2× lysis buffer (50 mM KCl, 100 mM Tris-HCl pH 7.4, 40% glycerol, 0.25% Triton X-100) supplemented with 40 mU per µl RNase Inhibitor for 10 min at room temperature. Lysed virus samples were diluted 1:10 in dilution buffer (5 mM (NH_4_)_2_SO_4_, 20 mM KCl, 20 mM Tris-HCl pH 8). Ten microliters of diluted virus sample and 10 µl of a serial dilution of virus standard (HEK293T cell supernatant of pCHIV at 5.088 × 10^9^ pU per µl) were mixed with 10 µl 2× reaction buffer (1× dilution buffer, 10 mM MgCl_2_, 2× BSA, 400 µM each dATP, dTTP, dGTP, dCTP, 1 pmol each RT forward and reverse primer, 8 ng MS2 RNA, SYBR Green 1:10,000) supplemented with 0.5 U of GoTaq Hotstart Polymerase. RT-PCR reactions were performed and read in a real-time PCR detector (CFX 96, Biorad) using the following program: (1) 42 °C for 20 min; (2) 95 °C for 2 min; (3) 95 °C for 5 s; (4) 60 °C for 5 s; (5) 72 °C for 15 s; 80 °C for 7 s; repeat 3–6 for 40 cycles, (7) melting curve. Primer sequences were the following: RT fwd primer: TCCTGCTCAACTTCCTGTCGAG, RT rev primer: CACAGGTCAAACCTCCTAGGAATG.

### Infectivity assay using TZM-bl reporter cells

Subconfluent TZM-bl cells were infected with serial dilutions of virus stocks or virus containing culture supernatant. After 48–72 h, cells were fixed with 3% PFA/PBS and incubated with β-Gal supplemented with 200 µg ml^−1^ X-Gal for subsequent counting of blue cells. Relative infectivity was calculated by dividing the infectivity by the used RT activity as determined by SG-PERT (see above).

### Spin-infection of primary T cells

Activated PBMC (2 × 10^5^ cells well^−1^) were resuspended with concentrated virus yielding 2 × 10^4^ blue cell units (MOI = 0.1) in a final volume of 50 µl in a 96-well plate (U-bottom or V-bottom) in the presence of 4 µg ml^−1^ Polybrene. Plates were centrifuged at 2000 rpm for 90 min at 37 °C and transferred to the incubator for 4–6 h. Polybrene was washed out and cells were cultured for 72 h in RPMI medium supplemented with 10 ng ml^−1^ IL-2 before analysis or setting up of collagen cultures.

### Sorting of NL4.3.Disp.YFP infected human T cells

Activated and CD8-depleted PBMC were sorted 72 h post infection with pNL4.Disp.YFP. Cells were resuspended in cold MACS buffer (PBS, 2 mM EDTA, 0.5% inactivated FCS) and mixed with anti-GFP beads (1:30, Miltenyi Biotec, 130-091-125) for 15 min in the refrigerator. Unbound beads were removed by centrifugation and infected cells were purified twice using MS columns within an OctoMACS magnet (both Miltenyi Biotec) according to the manufacturer’s instructions. Isolation efficiency was monitored by flow cytometry on samples collected during isolation. Typically, this procedure yielded 90–96% positive cells after the second magnetic column.

### Flow cytometry

To compare infection kinetics and depletion in collagen and suspension cultures, cultures were first treated with collagenase I (100 U, Worthington) for 30 min at 37 °C to yield cell suspensions. Cells were washed in PBS and stained with fixable viability dye 450 (eBioscience, 1:1000 in PBS) for 30 min at 4 °C, were washed in MACS buffer (PBS, 2 mM EDTA, 0.5% inactivated FCS) and subsequently stained with anti-CD8-PE Vio770 (1:100, Miltenyi Biotec, 130-096-556) and anti-CD3-PE (1:100, biolegend, 317308) for 30 min at 4 °C. After washing, cells were fixed in 3% PFA/PBS for 90 min. To detect intracellular p24, cells were permeabilized and stained with anti-p24-FITC (1:100 KC57 Beckmann Coulter, 6604665) in 0.1% Triton-X-100/PBS for 30 min at 4 °C. Cells were washed in MACS buffer and for absolute cell quantification cell counting beads (biolegend) were added prior to analysis with a FACSVerse (BD) and FlowJo software (Tree Star). CD4 T cells were identified as CD3 positive/CD8 negative cells. Relative depletion was calculated by correlating the frequency of CD4 T cells in the respective sample to the frequency of CD4 T cells in T20 controls, which was set to 100%.

### Proliferation assay by PKH26 dye dilution

For proliferation assays in parallel to infection kinetic experiments, a fraction of cells was stained with PKH26 in Diluent C (both Sigma Aldrich), according to manufacturer’s instructions (2.5 × 10^7^ cells in a total volume of 4 ml Diluent C with 2 µM PKH26 for 2 min at room temperature). Labeling reaction was stopped by adding equal volumes of heat-inactivated FCS for 1 min, followed by centrifugation and further washing steps in serum containing medium. Cells were then cultured in suspension or collagen for 0, 2, 4, and 7 days. At these time points cells were processed for flow cytometry (viability dye, CD8 PE-Vio770, CD3 PE, no permeabilization, see above), fixed and analyzed with FACSVerse. The sample at day 0 served to verify uniform labeling and to identify the parent population. Generations were modeled using ModFit LT (Verity Software).

### Generation of 3D collagen gels

Cell-containing (1 × 10^5^ cells per 100 µl gel) collagen gels were prepared as described^22^. Dense collagen gels (3.3–4.6 mg ml^−1^) were prepared by mixing highly concentrated rat tail collagen I (BD) with bicarbonate-buffered MEM on ice (15 µl 10× MEM, 17 µl 7.5% NaHCO_3_ (both Gibco) and 120 µl rat collagen I). This buffered collagen was mixed 1:1 with cells (2 × 10^6^ cells per ml media, yielding a final concentration of 1×10^5^ cells per 100 µl gel), was transferred to 96 wells (100 µl well^−1^) for long-term cultures or ibidi angiogenesis slides (10 µl well^−1^) for microscopy and allowed to polymerize within 30 min at 37 °C. Loose collagen gels (1.6 mg ml^−1^) were prepared correspondingly by mixing 750 µl bovine collagen I (PureColl, Nutacon) with bicarbonate-buffered Mem (50 μl 7.5% NaHCO_3_ and 100 μl 10× MEM) and combining it 2:1 with cells (3 × 10^6^ cells per ml media, yielding again a final concentration of 1 × 10^5^ cells per 100 µl gel mix) and allowed to pre-polymerize at 37 °C for 5 min depending on the volume used. The collagen-cell mix was transferred to the respective well format and allowed to polymerize at 37 °C within 30–45 min. Polymerized gels were overlaid with pre-warmed medium (RPMI 1640, FCS, PenStrep, 10 ng ml^−1^ IL-2). Where indicated, both gels and this supernatant were further supplemented with fusion inhibitor T20 (100 µM, Roche).

### Confocal reflection microscopy of collagen gels

Collagen matrix architecture was visualized by confocal reflection microscopy using the 488 nm laser and ×40 oil immersion objective of a Leica TCS SP5 microscope. A 10 µm stack of optical sections (z-stack) was acquired in 0.5 µm slices, which were subjected to maximum projections using the LAS AF software.

### Virus diffusion in 3D collagen

Fluorescent viral particles (HIV^GFP^ or HIV^mcherry^) generated by co-transfection of HEK293T cells with pCHIV and pCHIV^EGFP^ or pCHIV^mCherry^^54^ were incorporated into loose or dense collagen or suspended in medium. Movement of viral particles was recorded by a spinning disc microscope (Ultra-View ERS-6 spinning disc confocal microscope, Perkin Elmer) at 37 °C at maximum speed (200 ms) for 5 min and was automatically tracked (see below).

### Virus particle tracking and motility analysis

Automatic tracking of multiple fluorescent HIV-1 particles was performed using a probabilistic particle tracking approach which is based on Bayesian filtering and probabilistic data association^55^. This approach exploits multiple measurements and combines Kalman filtering with particle filtering. For particle detection, a Laplacian-of-Gaussian filter was used. The tracking method yields trajectories of individual HIV-1 particles.

Based on the computed trajectories, the motility of HIV-1 particles under different 3D collagen conditions and suspension was analyzed and the motion type was determined. We performed a mean square displacement (MSD) analysis^56^. MSD was computed as a function of the time interval Δ*t* for each trajectory of a tracked HIV-1 particle with a minimum time duration of 0.8 s (corresponding to five time steps). The MSD functions for all trajectories under one condition were averaged. An anomalous diffusion model ${\mathrm{MSD}} = 4\Gamma \Delta t^\alpha$ was fitted to the calculated MSD values which yielded the anomalous diffusion exponent *α* and the transport coefficient $\Gamma \left[ {{\upmu {\mathrm{m}^2}}\,{\mathrm{s}}^{ - \alpha }} \right]$^56^. We used MSD values from $0{\mathrm{s}} \le \Delta t \le 7{\mathrm{s}}$. The motion was classified into confined diffusion (*α* ≤ 0.1), obstructed diffusion (0.1 < α < 0.9), and normal diffusion (*α* ≥ 0.9)^57^. We also fitted the normal diffusion model ${\mathrm{MSD}} = 4\Gamma \Delta t$^56^ to the MSD values to determine the diffusion coefficient D = Γ[μm^2^s^−1^].

In addition, based on the tracking result, events of particle interaction with the collagen structure were identified (sticking events). For all tracked HIV-1 particles the velocities were computed and represented in a velocity histogram. From the histogram we determined a velocity threshold of $v_{{\mathrm{Th}}} = 1.0\;\upmu {\mathrm{m}}\;{\mathrm{s}}^{ - 1}$ to distinguish different subpopulations. The interaction time of an HIV-1 particle with the collagen structure was calculated as the time duration for which a particle yielded velocities below *v*_Th_. For a consistent comparison of interaction times between different collagen conditions, we considered trajectories with a time duration of up to 15 s (corresponding to 76 time steps).

### Live-cell imaging in 3D collagen

Cells infected with HIV-1 NL4.3DispYFP were purified by magnetic isolation as described above. Control CD4 cells from uninfected cultures were purified correspondingly with anti-CD4 magnetic beads (Miltenyi Biotec, 130-045-101). Infected and uninfected cells were either embedded separately in collagen or were stained with PKH cell dyes as for proliferation assays to enable distinction between infected (green, PKH67) and target cells (red, PKH26) in cocultures. Cells within collagen gels were monitored in angiogenesis slides (ibidi) using bright field, and for stained cells, green and red channels of an inverse light microscope (×10 objective, Nikon Ti-E), equipped with climatisation control maintaining 37 °C and 5% CO_2_ (Perkin Elmer). For the duration of up to 3 h, micrographs were taken in 30 s to 1 min intervals.

### Cell segmentation and tracking

To obtain cell trajectories we applied the tracking-by-detection method available in ilastik^58^. In this tracking-by-detection method, the cells need to be segmented first. To do so, we employ a convolutional neural network to predict foreground probabilities for each pixel. More specifically, we use a U-net^59^ in its standard configuration using four stages of pooling to downsample, pixel replication for upscaling, skip connections, dropout in the convolutional layers, ReLU activation functions and batch normalization. We trained it to use the three available channels as input, using 10 manually annotated images as ground truth. Training data were augmented by applying elastic transformations. We ran 129 epochs of training until the loss seemed to have converged, and the resulting weights are employed to predict the foreground probability of all frames in all videos. The cell segmentation hypotheses are then given as connected components after thresholding these foreground probabilities.

Cell trajectories are reconstructed by finding the most probable configuration of a graphical model spanning the whole video^60^ where detections are assigned to detections in adjacent frames. To deal with occasional segmentation errors, where two seemingly overlapping cells are falsely combined in a single segment, the used tracking model allows for detections to be shared between multiple object tracks. Local evidence about these merged detections is injected into the model via an object count classifier that predicts the number of objects contained in every segment based on intensity and shape features computed from raw data and segmentation. The object count classifier itself was trained by manually annotating a few instances of single cells and mergers of different sizes on two datasets, and then applied to all videos. By solving an integer linear program, we find the globally most probable configuration of how many cells are contained in every segment and where they move in consecutive frames. In a post-processing step, any merged detections are split using a Gaussian mixture model, with the number of components given by inference in the probabilistic graphical model.

The tracking results are then exported to the ImageJ-plugin MaMuT^61^ for manual corrections of the tracks, where also cell identity (infected if green, not-infected if red) is manually annotated.

### Track analysis

Cell segmentation, automated tracking and subsequent manual track correction in the ImageJ-plugin MaMuT can for some tracks result in the identification of multiple spots per cell and time frame. Therefore, we first calculated center-of-mass coordinates. Based on this data we calculate the instantaneous cell velocity, the fraction of mobile cells, the arrest coefficient (between 0 and 1 for a cell which is mobile or immobile for all time steps, respectively), the confinement ratio, the mean turning angle, and the mean squared displacement (MSD)^3^. As a threshold to classify cells as mobile, we used 2 µm min^−1^ instantaneous velocity, as a well-accepted threshold in the field of lymphocyte migration^62^. To identify cell–cell contacts, we computed the convex hull as an estimator for the cell contour for each cell track at each time point. A cell–cell contact is then defined by mutually overlapping convex hulls at a given time point. From these data we then extracted contact frequency (fraction of time spent in contact with another cell) and partner frequency (how many different cells are contacted per time). Since the cells migrate in 3D collagen environments, but we record only a 2D projection as a single plane in wide-field microscopy, we filtered out false positive cell–cell contacts that are displaced in *z*-direction. We tried various approaches and found the difference between maximal intensity values of cells to be a good discriminator to detect such false positives. The threshold for this difference was manually evaluated and contacts exceeding this threshold were excluded as false positive contacts from further analysis.

### Modeling of cell proliferation and infection dynamics

We used a stepwise approach to analyze the experimental data for determining the contribution of cell-free and cell-associated transmission to viral spread in the different environments. Mathematical models of increased complexity based on ordinary differential equations (ODE) were combined with corresponding experimental measurements in order to quantify cell proliferation, viral turnover, and the infection dynamics.

### Quantifying T-cell proliferation in uninfected cultures

Transferred CD8, *T*_CD8_, and CD4, *T*_CD4_, positive T cells are assumed to die with rates *δ*_CD8_ and *δ*_CD4_, respectively, and to undergo competing proliferation with maximal proliferation rates *λ*_CD8_ and *λ*_CD4_ until a total capacity of *T*_c_ cells is reached. In addition, an adaptation phase, *τ*, which was fixed to 2.5 days, was included that accounts for the time transferred cells need to adapt to their new environments at the start of each experiment. During this time cells are lost according to their corresponding death rates. T-cell dynamics within the different environments can then be described by the following system of ordinary differential equations:

$$\begin{array}{l}\frac{{dT_{{\mathrm{CD}}8}}}{{dt}} = \lambda _{{\mathrm{CD}}8}T_{{\mathrm{CD}}8}\left( {1 - \frac{{T_{{\mathrm{CD}}8} + T_{{\mathrm{CD}}4}}}{{T_{\mathrm{c}}}}} \right) - \delta _{{\mathrm{CD}}8}T_{{\mathrm{CD}}8}\\ \frac{{dT}}{{dt}} = \lambda _{{\mathrm{CD}}4}T\left( {1 - \frac{{T_{{\mathrm{CD}}8} + T_{{\mathrm{CD}}4}}}{{T_{\mathrm{c}}}}} \right) - \delta _{{\mathrm{CD}}4}T\\ {\mathrm{with}}\;\lambda _{{\mathrm{CD}}8} = \lambda _{{\mathrm{CD}}4} = 0\;{\mathrm{for}}\;{\mathrm{t}} \,< \, {\mathrm{\tau }}.\end{array}$$

For parameter estimation, obtained T cell counts at day 0 were used as initial conditions.

### Quantification of virus dynamics under T20 treatment

We used a model accounting for non-productively, *I*_NP_, and productively infected cells, *I*_P_, virus in culture, *V*_c_, and the supernatant, *V*_s_, to describe virus dynamics after T20 treatment. As treatment by T20 is assumed to block any new infections, non-productively infected cells either die at the same rate as uninfected cells, *δ*_CD4_, or turn into productively infected cells with rate *κ*_*I*_. Productively infected cells are assumed to have an average productive life span of 1/*δ*_*I*_ and release new virions into the culture with a viral production rate *ρ*. Virions diffuse to the supernatant with a diffusion rate *κ*_v_ and are cleared from the system at rate *c*_v_, which is fixed to *c*_v_ = 0.44 day^−1^ to correspond to the half-life of RT activity of 38 h as evaluated experimentally. With this, infection dynamics after T20 treatment is then described by the following system of differential equations:

$$\begin{array}{l}\frac{{dJ}}{{dt}} = - \kappa _IJ - \delta _{{\mathrm{CD}}4}J\\ \frac{{dI}}{{dt}} = \kappa _IJ - \delta _II\\ \frac{{dV_{{c}}}}{{dt}} = \rho I - c_{{v}}V_{{v}} - \kappa _{{v}}V_{{c}}\\ \frac{{dV_{{s}}}}{{dt}} = \kappa _{{v}}V_{{c}} - c_{{v}}V_{{s}}\end{array}$$

For parameter estimation, *δ*_CD4_ was fixed to the best estimate for the death rate of uninfected CD4 cells from the experiment in the absence of infection, and *κ*_*I*_ was set to 1.39 day^−1^ corresponding to a half-life of cells in eclipse phase of 12 h. To account for the change of media in collagen environments, viral concentration in the supernatant was set to 0 at day 2, 4, 7, 9, 11, 14, 16, and 18. As change of media leads to mixing in liquid environments, viral concentration in culture and supernatant was halved at days of media change in the suspension environment. This leads to the frequent drops observed in the predicted viral concentration in Fig. 3e and is required to estimate a single cell-free transmission rate *β*_f_ across different environments by accounting for the differing viral concentrations before and after media change.

Experimental measurements for productively infected cells and virus in culture and supernatant at day 0 were taken as initial conditions. As non-productively infected cells cannot be detected by FACS-analysis, a proportion of non-infected target cells was estimated across all environments to define the initial concentration of non-productively infected cells. Infected cell counts after day 9 were not considered in the analysis, as cell numbers were indistinguishable from background levels determined from the proliferation data.

### Mathematical model for the complete infection dynamics

With the previous assumptions and descriptions, the complete mathematical model describing cell proliferation and infection dynamics for the co-transfer experiments of infected and uninfected cells (ODE-model) is given by

$$\frac{{dT_{{\mathrm{CD8}}}}}{{dt}} = \lambda _{{\mathrm{CD8}}}T_{{\mathrm{CD8}}}\left( {1 - \frac{{T_{{\mathrm{CD8}}} + T + T_{{\mathrm{ref}}} + J + I}}{{T_{\mathrm{c}}}}} \right) - \delta _{{\mathrm{CD8}}}T_{{\mathrm{CD8}}}$$

$$\frac{{dT_{{\mathrm{ref}}}}}{{dt}} = \lambda _{{\mathrm{ref}}}T_{{\mathrm{ref}}}\left( {1 - \frac{{T_{{\mathrm{CD8}}} + T + T_{{\mathrm{ref}}} + J + I}}{{T_{\mathrm{c}}}}} \right) - \delta _{{\mathrm{CD4}}}T_{{\mathrm{ref}}}$$

$${\frac{{dT}}{{dt}} = \left( {\lambda _{{\mathrm{CD4}}}T + \left( {\lambda _{{\mathrm{CD4}}} - \lambda _{{\mathrm{ref}}}} \right)T_{{\mathrm{ref}}}} \right)\left( {1 - \frac{{T_{{\mathrm{CD8}}} + T + T_{{\mathrm{ref}}} + J + I}}{{T_{\mathrm{c}}}}} \right) - \beta _fTV_i - \beta _{\mathrm{c}}TI - \delta _{{\mathrm{CD4}}}T}$$

$$\frac{{dJ}}{{dt}} = \beta _fTV_i + \beta _{\mathrm{c}}TI - \kappa _IJ - \delta _{{\mathrm{CD4}}}J$$

$$\frac{{dI}}{{dt}} = \kappa _IJ - \delta _I{\mathrm{I}}$$

$$\frac{{dV_{{i}}}}{{dt}} = f_{{i}}\rho I - c_{{i}}V_{{i}} - c_{{v}}V_{{i}} - \kappa _{{v}}V_{{i}}$$

$$\frac{{dV_{{n}}}}{{dt}} = (1 - f_{i})\rho I + c_{{i}}V_{{i}} - c_{{v}}V_{{n}} - \kappa _{{v}}V_{{n}}$$

$$\frac{{dV_{{s}}}}{{dt}} = \kappa _{{v}}(V_{{i}} + V_{{n}}) - c_{{v}}V_{{s}}$$

Here, CD8 cells, *T*_CD8_, and an additional population of refractory cells, *T*_ref_, cannot get infected, but affect the proliferation capacity of target cells. The proliferation of target cells is determined based on the proliferation of refractory cells, *λ*_ref_, and the expansion rate of the total CD4 population, *λ*_CD4_. In addition, we distinguish between infectious, and non-infectious virus in the culture, with only a fraction, *f*_i_, of virus produced by productively infected cells being infectious. Furthermore, infectious virus loses its infectivity with rate *c*_i_, which is fixed such that the half-life of infectious virus is 17.9 h^63^. To account for the two different transmission modes, activated CD4 cells can get infected either proportional to the concentration of infectious virus in the culture, *V*_i_, with rate *β*_f_ (cell-free transmission) or proportional to the number of productively infected cells, *I*, at rate *β*_c_ accounting for cell–cell transmission.

The model was fitted to estimate *β*_c_ and *λ*_ref_ for each of the different environments. In addition, one transmission rate for cell-free infections, *β*_f_, was estimated across all environments where *β*_f_ was reduced to 14% in collagen compared with suspension as evaluated experimentally (Fig. 2g). All other parameters were defined as described above or obtained from the previous analyses quantifying T-cell proliferation and virus dynamics during T20 treatment. Measurements for CD8, target and productively infected cells, as well as viral concentrations at day 0 were taken as initial conditions. As before, the initial proportion of non-infected target cells and refractory cells, as well as the proportion of infectious virus was estimated across all environments, as these values could not be measured.

### Parameter estimation

All models were fitted to the data using the *optim*-function in the *R-*language of statistical computing^64^. To avoid bias in parameter estimates due to reduced recovery of cells from collagen environments (only 55% of cells compared with suspension are recovered), cell counts from collagen cultures were normalized by multiplying each measurement by a factor of 1.8. Models were fitted to the mean values of three independent experiments to diminish experimental variance in the data using a maximum likelihood estimator that assumed normally distributed errors. The experimental variance in the data was neglected and a relative measurement error was estimated for T cells and viral load. Parameter identifiability was assessed based on a profile likelihood approach^65^.

### Cellular Potts model

To simulate migrating cells in a 2D collagen environment, reflecting live-cell imaging videos, a cellular Potts model (CPM) formalism was applied using the software Morpheus^66^. The CPM models cellular behavior in a grid-based environment with several connected grid sites defining a single cell^36^. Each cell is characterized by a specific cell type, *τ*, and a unique identifier, *σ*, as well as its grid position defined by the coordinates *u* and *v*. Movement of cells is guided by stochastic membrane fluctuations that can result in a shift in position if a global energy function ${\cal{H}}$ is minimized^67^. This global energy function ${\cal{H}}$, termed Hamiltonian, consists of a term describing the sum over all surface energies *J*, as well as volume and perimeter constraints per cell. The surface energy *J* (also denoted as *J*-value) is defined by the adhesion of each cell to its surrounding cell types or media. The surface energy of each cell is multiplied with the term (${1}\, -{\delta}_{{\sigma}_{u},{{\sigma}_v}}$), where *δ* defines the Kronecker-delta (${\delta}_{{\sigma}_{u},{\sigma}_{v}}$ *=* 1 if *σ*_*u*_ *=* *σ*_*v*_ and 0 otherwise) to consider only interactions between different cells. Volume and perimeter constraints ensure that cells try to maintain their size. The constraints are defined by the squared difference between the current cell volume or perimeter (*a*_*σ*_, *p*_*σ*_) and the target cell volume or perimeter (*A*_*σ*_, *P*_*σ*_), respectively. The terms are multiplied by a Lagrange multiplier *λ*, determining the strength of the constraint^68^. The formula for ${\cal{H}}$ therefore results as

$${\cal{H}} = \mathop {\sum}\limits_{u,v} {J_{\tau (\sigma _u),\tau (\sigma _v)}(1 - \delta _{\sigma _u,\sigma _v})} + \mathop {\sum}\limits_\sigma {\lambda _{{\mathrm{Area}}}(a_\sigma - A_\sigma )^2} + \mathop {\sum}\limits_\sigma {\lambda _{{\mathrm{Perimeter}}}(p_\sigma - P_\sigma )^2}$$

To simulate cell motility, at each simulation step a grid site within the 2D lattice is chosen at random. For this selected grid site, one of the eight neighboring grid sites not belonging to the same cell is chosen at random for a possible shift in position. If the shift decreases the global energy, the site is copied to this random position^36^, otherwise the copy attempt is accepted with the Boltzmann probability of $e^{ - {\mathrm{\Delta }}{\cal{H}}/T}$. Thereby, the parameter *T* defines the membrane fluctuation amplitude of cells for exploring the neighborhood. Target and infected cells are assumed to be motile with both cell types following persistent motion. Persistence is characterized by the stability to keep the direction of movement and a memory of this direction (direction-update interval), meaning each cell is more likely to follow a path close to its current direction. Persistent motion is implemented into the CPM by extending $\Delta {\cal{H}}$ by ${\mathrm{\Delta }}{\cal{H}}\prime = {\mathrm{\Delta }}{\cal{H}} - \mu {\mathrm{cos}}(\alpha ),$ with *α* being the angle between the target and considered direction^3^. Therefore, a copy attempt to a new lattice site is likely to be accepted if *α* is small.

### Simulation environment and default parameters

We simulate a total area of 800 × 800 μm^2^ with each grid site of the lattice having a length of 1 μm. Each grid point in the lattice is surrounded by eight neighbors, following Moore-neighbor conditions. In addition, we assume periodic boundary conditions with cells leaving at one side of the grid reentering at the opposite side. Our simulation distinguishes between infected and uninfected T cells, collagen particles and free space. T cells were defined with a target area of *A*_T_ = 100 μm^2^ and a connectivity constraint^69^ that prevents cells from breaking apart. While infected and uninfected T cells were considered as being motile, collagen particles were implemented motionless but deformable. For loose collagen simulations, we used a total number of 14,400 collagen particles with a target area of *A*_C_ = 20 μm^2^ and a target perimeter of *P*_C_ = 0.2 μm. The energy between collagen particles (J_CC_) collagen and medium (J_CM_) and in medium (J_MM_) was set to 0 to allow the formation of collagen structures^68^.

In our simulations, one simulation step corresponds to 1 s in real time. Cell positions and cell-to-cell contacts were recorded every 30 s according to the frame rate used for live-cell imaging. A cell-to-cell contact is registered when two adjacent cells share one lattice site. For each simulation, an initial phase of 200 s is run before measurements are taken to allow the system to reach steady conditions.

### Parameterizing cell motility

To parameterize the motility of infected and uninfected cells in our CPM, we adjusted our model based on measurements from the time-lapse image analysis following both cell types for 1 h in loose collagen environments. We used the computational parallelization and high-performance approach “pyABC”^4,70^ to automatically adjust simulation parameters to experimental data. The computational pipeline overcomes the problem of statistical interference for parameter fitting in stochastic multi-scale models by using a parallel approximate Bayesian computation sequential Monte Carlo (pABC-SMC) algorithm^4^. The pyABC workflow tests multiple parameter sets in parallel by subsequently minimizing distance measurements between experimental and simulated data. To this end, each simulation is evaluated using the R Motilitylab package (http://www.motilitylab.net) to calculate the mean square displacement, the velocity, mean turning angle, straightness and arrest coefficient of each infected or uninfected cell (see above for the calculation of these quantities). The distance between simulations and in vitro measurements was then determined based on a weighted sum of least-squares for these quantities. To account for differences in measurement scales of velocity, mean turning angle, straightness and arrest coefficient, we calculated the coefficient of variance, which gives a standardized variance with the ratio between standard error and mean. The squared distance of coefficients of variance between experimental and simulated data was added to the least-square distance, *d*_i_, for each quantity *i* resulting in the distance measure

$$d_{i} = \frac{{(\mu_{i} - u)^2}}{{\sigma ^2}} + \left( {\frac{\sigma_{i} }{\mu_{i} } - \frac{s}{u}} \right)^2$$

Here, *μ*_i_ and *σ*_i_ are the mean and standard deviation, respectively, of the experimental data, and *u* and *s* the corresponding values of the simulations. The total sum of least-squares defining the distance between simulated and experimental data is then given by

$$d_{{\mathrm{Sim}} - {\mathrm{Exp}}} = 0.01 \times d_{{\mathrm{MSD}}} + d_{{\mathrm{speed}}} + d_{{\mathrm{MTA}}} + d_{{\mathrm{straight}}} + d_{{\mathrm{arrest}}}$$

The weighting factor of 0.01 for the MSD distance was chosen to ensure that all different quantities were weighted equally. Because the MSD can reach values from 0 to 10^5^ μm^2^ s^−1^, the calculated distances for this quantity become large at later time points, rendering the contribution of all other motility measurements to the total distance negligible. The obtained parameter sets resulting in the smallest distance between experimental and simulated cellular motilities are shown in Supplementary Table 1.

### In silico spatial infection model

To analyze long-term infection courses and investigate cell-to-cell transmission on a single-cell level, we extended our CPM by including the processes of infection, viral replication, cell proliferation, and death parameterized with values obtained by our ODE-model. Infection was implemented allowing cell-to-cell transmission from infected to uninfected target cells. An infectious cell-to-cell contact was defined by a minimal contact duration, *D*_min_, between the two cells. The contact duration, *D*, between two cells increases incrementally as long as the contact is not broken. A contact is considered as broken if no lattice site is shared between two cells, resetting *D* = 0. If *D* *>* *D*_min_, an infection event was triggered. Upon infection, the newly infected cell entered an eclipse phase for on average 1/*κ*_I_ = 17.3 h to account for viral integration and replication, in which the cell keeps the target cell motility characteristics and is not yet productively infectious. At the end of this phase, the cell turns into an infected cell with the ability to infect other target cells. Here, we studied a multiple infection model, i.e., with an infected cell able to infect several CD4 target cells simultaneously if a minimal contact duration between the cells is fulfilled. To match the experimental conditions, we additionally considered a population of CD8 T cells in the simulations that followed the same motility characteristics as uninfected CD4 T cells, as well as a fraction of refractory CD4 T cells. Accounting for cellular turnover, both cell populations are considered to proliferate and die during the time-course of a simulation. As before, we assume density dependent proliferation of CD4 and CD8 T cells with the actual proliferation rates calculated at each time step by

$$\lambda _X^ \ast = \lambda _X\left( {1 - \frac{N}{C}} \right)$$

Here, *λ*_X_ defines the maximal proliferation of the specific cell type, *N* the actual total number of cells in the grid, and *C* the carrying capacity of the grid in number of cells. Given loose collagen conditions and using the standard cell concentration, the simulated grid can hold a maximum of *C* = 1200 cells with still 37.5% free space, which is comparable with values obtained by confocal reflection microscopy. At each time step, for each cell a division event is then determined with probability $1 - e^{ - \lambda _{\mathrm{X}}}$. We assume that infected CD4 T cells do not divide, and that cell division only occurs after an initial adaptation phase. Similar to cell proliferation, the probability of a cell to die is calculated at each time step by $1 - e^{ - \delta _{\mathrm{X}}}$, with *δ*_X_ defining the constant death rate of the corresponding cell population, i.e., infected CD4, refractory, and uninfected CD4 and CD8. Cells in eclipse phase are assumed to keep the same death rate as uninfected CD4 cells. For our simulations, we used the rates for cell proliferation (*λ*_CD4_ = 0.78 d^−1^, *λ*_CD8_ = 0.58 d^−1^) and death (*δ*_CD4_ = 0.23 d^−1^, *δ*_I_ = 0.48 d^−1^, *δ*_CD8_ = 0.11 d^−1^), as well as the duration of the adaptation phase (*τ* = 2.5 days), as determined by our analyses (see Table 1 in the main text).

### Considered cell concentrations and simulation structures

To adapt cell motility characteristics and to compare cell-to-cell contacts between simulated and experimental data, we simulated the dynamics using 150 uninfected and infected cells each, resulting in 50% free space as observed in the in vitro experiments. Cells were followed for 1 h with cell positions and cell-to-cell contacts recorded every 30 s, which corresponds to the frame rate used in the experiments. The infection process was neglected for these analyses.

With the obtained parameterization for the motility of infected and uninfected cells, the spread of infection was simulated for a total of 18 days. As a standard concentration, we used a total number of 300 cells comprising 57 (19%) CD8 T cells and 243 (81%) CD4 T cells, with 15 of these cells being infected which corresponds to the conditions in the experiments. According to our previous estimates based on our mathematical model, roughly 10% of the initial CD4 T cells (23 cells) were implemented to be refractory T cells not able to become infected. Experimentally, we observed a maximal fourfold increase of total cell numbers in the collagen cultures. Therefore, the carrying capacity in our simulations given the standard concentration was set to *C* = 1200 cells. Supplementary Table 2 shows the corresponding cell numbers for the individual cell populations used in the simulations considering 2-, 5-, or 10-fold higher cell concentrations at the beginning. As the maximal fold increase of the total cell population in a culture is regulated by the proliferation capacity of individual cells, the availability of nutrition in the media, as well as the spatial limitations of the culture, we assumed a saturated increase of the carrying capacity for increasing initial cell numbers.

### Quantification and statistical analysis

Statistical analysis was performed using GraphPad Prism. Statistical tests used are stated in the figure legends where they appear. Statistical significance of parametrically or not normally distributed datasets was analyzed by Student’s *t* test or Mann–Whitney *U* test, respectively. ns: not significant; *p-value < 0.05; **p-value < 0.01; ***p-value < 0.001.

### Reporting summary

Further information on research design is available in the Nature Research Reporting Summary linked to this article.

## Supplementary information

Supplementary Information

Peer Review File

Reporting Summary

Description of Additional Supplementary Files

Supplementary Movie 1

Supplementary Movie 2

Supplementary Movie 3

Supplementary Movie 4

## Data Availability

The data supporting the findings of this study are available within the article, in supplementary files, and upon reasonable request from the corresponding author.

## Code availability

Segmentation was based on a U-Net^59^ trained to the specific data set with the original architecture available at https://lmb.informatik.uni-freiburg.de/people/ronneber/u-net/. The complete code for conservation tracking^60^ including a structured learning workflow is available open source at http://ilastik.org. The ODE-model and CPM were developed using the open source software R (ref. ^64^) and Morpheus (ref. ^66^, https://imc.zih.tu-dresden.de/wiki/morpheus/), respectively. All aspects concerning the specific protocols and the parameterizations for the mathematical models are defined within the text and Methods (see e.g., Table 1, Supplementary Tables 1 and 2). All individual software codes are available upon reasonable request from the authors.
