# Supplementary material for: Experimental and computational analyses reveal that environmental restrictions shape HIV-1 spread in 3D cultures
Source: Nat Commun. 2019 May 13;10:2144. doi: 10.1038/s41467-019-09879-3 (PMC6514199; doi:10.1038/s41467-019-09879-3)
Supplement: Supplementary file 1 — Supplementary Information [file 41467_2019_9879_MOESM1_ESM.pdf]

Supplementary Information

Experimental and computational analyses reveal that environmental restrictions shape HIV-1 spread in 3D cultures

Andrea Imle, Peter Kumberger, Nikolas D. Schnellbächer, Jana Fehr, Paola Carrillo-Bustamante, Janez Ales, Philip Schmidt, Christian Ritter, William J. Godinez, Barbara Müller, Karl Rohr, Fred A. Hamprecht, Ulrich S. Schwarz, Frederik Graw and Oliver T. Fackler

Supplementary Table 1.: Model parameters adjusted with pyABC. 14 parameters were adjusted with pyABC. The best estimate for each parameter was retrieved from the parameters from pyABC that resulted in the smallest distance between cell motilities from experimental data and simulations.

Parameter	Symbol	Best estimate
**Persistent motion for cells**		
Persistence strength infected cells	PS_i	82.43
Persistence strength target cells	PS_t	152.07
Direction update interval infected cells	DT_i	83.66
Direction update interval target cells	DT_t	54.13
**Collagen**		
Resistance to compression	$\lambda_{Area,C}$	205.28
Membrane stiffness	$\lambda_{Perimeter,C}$	143.18
**Adhesion energy**		
Energy between target cells	J_{TT}	386.74
Energy between infected cells	J_{II}	91.13
Energy between target and infected cells	J_{TI}	299.59
Energy between target cells and medium	J_{TM}	326.81
Energy between target cells and medium	J_{IM}	341.05
Energy between target cells and collagen	J_{TC}	369.87
Energy between infected cells and collagen	J_{IC}	432.79
**CPM**		

Parameter	Symbol	Best estimate
Temperature	Temp	61.89

Supplementary Table 2: Initial number of cells and carrying capacity assumed for different concentrations of cells at the beginning. The carrying capacity C is determined based on the total initial cell number, N, and the assumed relationship $C(N) = C_{max}(1 - e^{-\lambda N})$. The parameter λ was estimated using the observed 4-fold increase for the standard concentration, C(300)=1200 cells, the assumption that for lower cell numbers only their proliferative capacity will determine the capacity, leading to C(150)=600 cells, and that for a 10-fold higher initial concentration cell growth is limited by the available space in the cell culture, i.e., leading to C(3000)=4000 cells.

Cell Type	1x	2x	5x	10x
**CD4 uninfected**	205	419	1067	2147
**CD4 refractory**	23	46	118	238
**CD4 infected**	15	15	15	15
**CD8**	57	120	300	600
**Carrying capacity, *C***	1200	2021	3367	4000

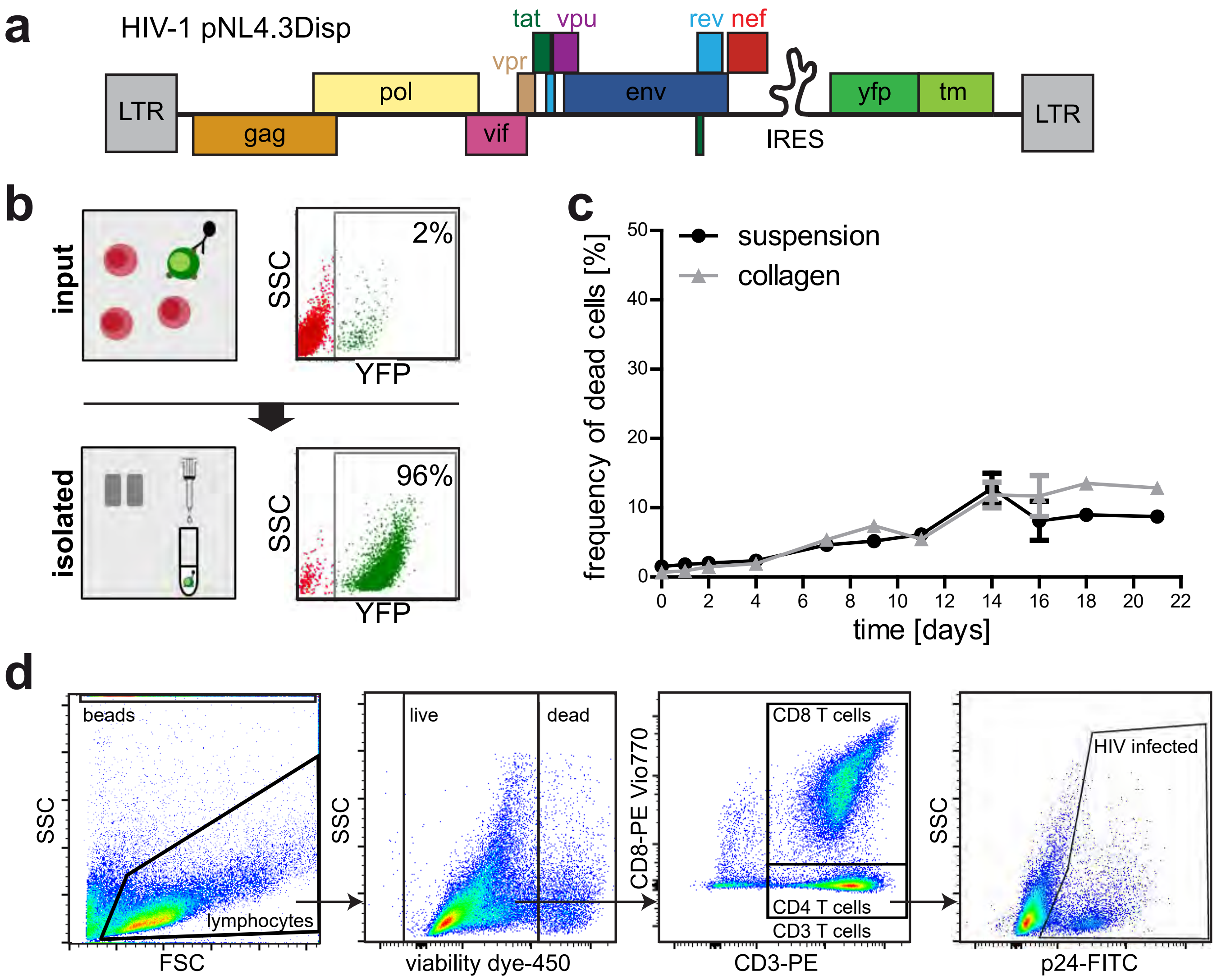

Imle et al. 2018 Supplementary Figure 1

Supplementary Figure 1. Sorting, and gating strategy.

(a) Scheme of sortable provirus (HIV-1 NL4.3 IRES.Display.YFP, HIV-1 NL4.3DispYFP). (b) Principle of purification of infected cells (green). Surface displayed YFP is detected by antibody-coupled magnetic beads, which enable purification by passage through magnetic column. Percentages indicate cell frequency in the respective gate. (c) Viability of lymphocytes in suspension and collagen over time. Fixable viability dye marks dead lymphocytes at indicated time points. The Fig. shows data from one representative out of 4 donors. Mean and standard deviation of triplicate measurement are shown. (d) Gating strategy. From left to right: lymphocyte gate and bead gate for absolute cell quantification displayed among all events. Lymphocytes are distinguished between dead and alive using a fixable viability dye. Live lymphocytes are categorized as CD3 T cells, which are further subdivided in CD8 T cells (CD3+, CD8+) and CD4 T cells (CD3+, CD8-). HIV-1 infected CD4 T cells are detected by anti p24-antibodies.

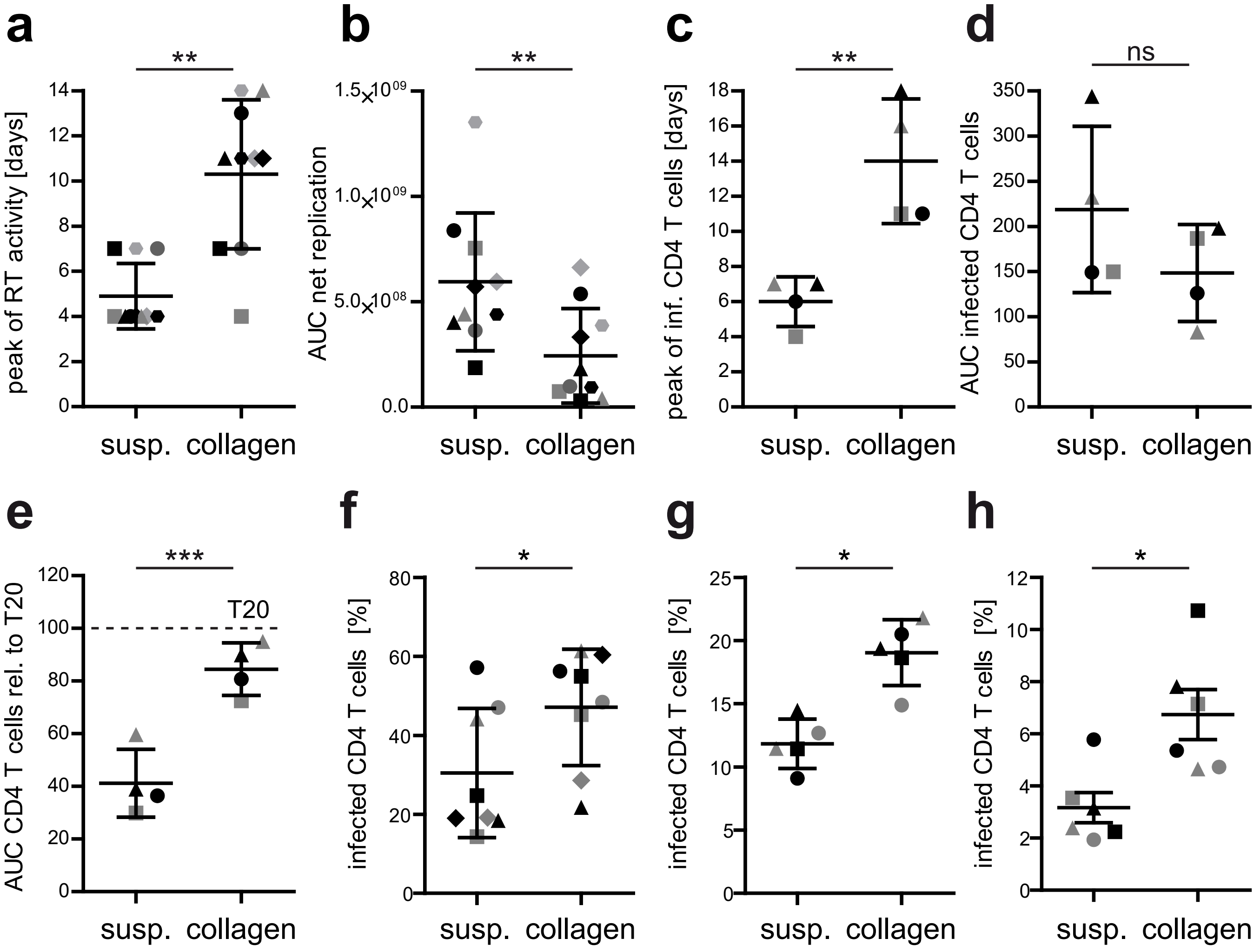

Imle et al. 2018 Supplementary Figure 2

Supplementary Figure 2. HIV-1 spread kinetics and permissivity to infection.

HIV-1 spread in suspension or collagen from multiple donors. (a) Day of maximum virus concentration obtained in cells from multiple donors designated by symbol and color. (b) Summary of virus concentration obtained in cells from multiple donors. To monitor net replication, area under the curve values (AUC) of virus concentration in supernatants of T20-treated cultures over 16 up to 21 days were subtracted from AUC values of the corresponding T20-negative samples. (c) Day of maximum percentage of infected CD4 T cells. (d) AUC of percentage of infected CD4 T cells. (e) AUC of residual CD4 T cells relative to respective T20, which was set to 100 (dashed line). Individual points indicate independent experiments using cells from individual donors. Statistical comparisons were based on paired t-test (f-h). (f) Permissivity after culture by cell-free infection. PBMCs were activated and cultured for 4-7 days in suspension or collagen. Subsequently, PBMCs were retrieved from culture by collagenase digestion, counted and spin-infected with HIV-1. After 72 h post infection, infected CD4 cells were quantified by p24-staining in flow cytometry. Individual data points represent means of duplicate to triplicate measurements from independent experiments with different donors. Mean +/- standard deviation, paired t-test. (g) Permissivity after culture by cell-cell infection. PBMCs were activated and either cultured in suspension or collagen. After 72 h, PBMCs were retrieved from suspension and collagen culture by collagenase digestion and counted. For subsequent cell-cell infection, cells were mixed 4:1 with donor-matched PBMCs which had been infected with HIV-1 for 72 h (stained with 5 µM CMTMR). Another 72 h later, infection of CMTMR-negative cells was analyzed by p24-staining in flow cytometry. Mean +/- standard deviation, paired t-test. (h) Permissivity in suspension and collagen culture by cell-free infection. Activated PBMCs were mixed at a MOI of 0.5 with single-round HIV-1 (env pseudotyped pNL4.3 Δenv) and incorporated into suspension or collagen. After 72 h, cells were retrieved by collagenase digestion, and infected CD4 cells were quantified by p24-staining in flow cytometry. Mean +/- standard deviation, paired t-test. ns: not significant; *: p-value<0.05; **: p-value<0.01; ***: p-value<0.001.

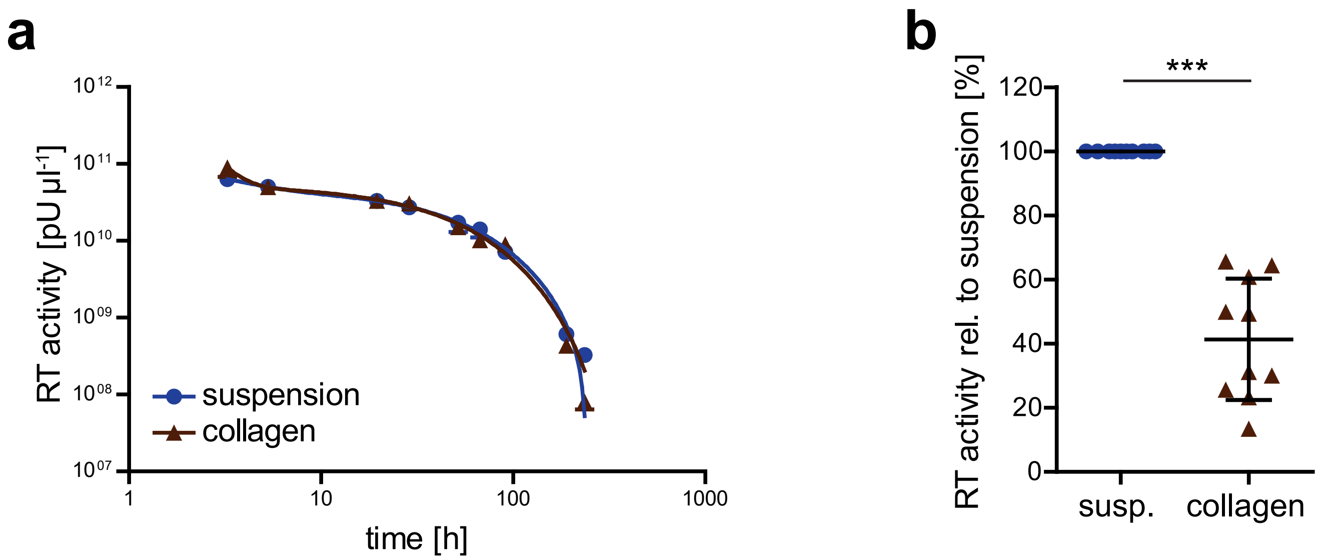

Imle et al. 2018 Supplementary Figure 3

Supplementary Figure 3. Collagen reduces virus production.

(a) Half-time of RT activity for virus embedded in medium (suspension, blue) or collagen (brown). At the indicated time points, total RT activity was quantified as the sum of virus in the supernatant and within the gel (upon collagenase digestion). A two phase decay exponential fit was used to model the data to account for fast decay of free RT from damaged particles and slow decay of RT from intact particles. One representative experiment out of three is shown. Data points indicate mean +/- standard deviation of slow halftimes from the three experiments. (b) Virus production in culture. 72 h upon spin-infection, HIV-1-infected PBMCs were incorporated into suspension or collagen in the presence of 100 µM T20 to inhibit further spread to target cells. Total virus production was quantified as the sum of SG-PERT signal from supernatants and collagenase digested cultures another 48-72 h later. Data were normalized relative to the results from the respective suspension experiment, which is set to 100%. Individual symbols indicate independent experiments with cells from individual donors. Mean +/- standard deviation, paired t-test was used for statistical analysis.

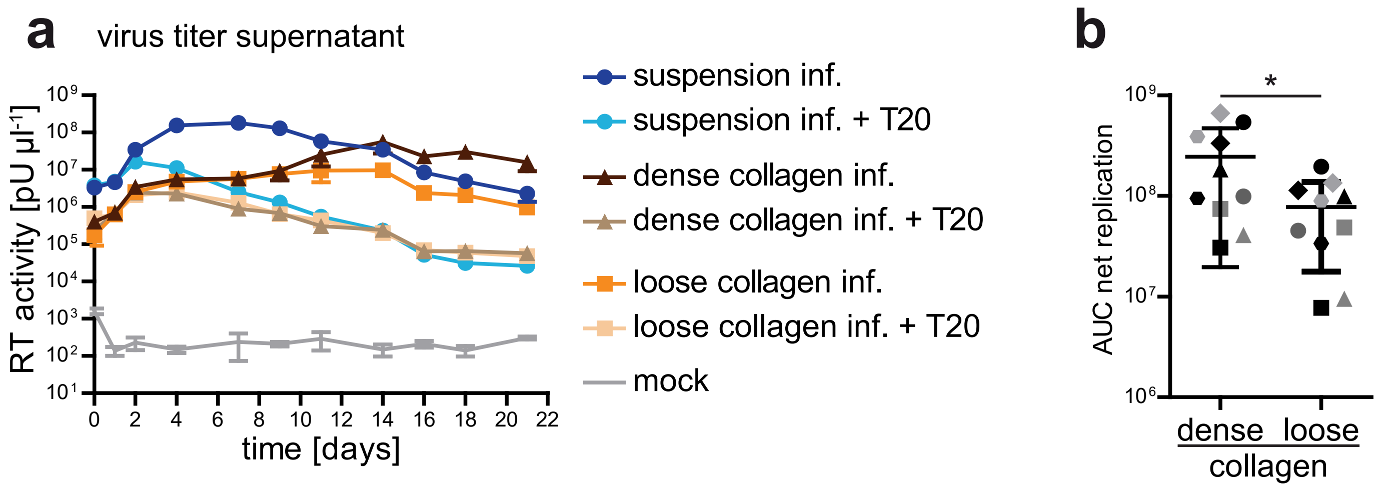

Imle et al. 2018 Supplementary Figure 4

Supplementary Figure 4. 3D confinement dictates HIV-1 spread efficiency

(a) Cells from a representative donor showing HIV-1 spread in suspension, dense and loose collagen over time including respective T20 controls and mock. Virus titers determined from supernatants by SG-PERT. Mean and standard deviation from triplicate measurements are shown. (b) Summary of virus titers obtained in cells from multiple donors designated by color and symbol. To monitor net replication, AUC values of virus concentration in supernatants of T20-treated cultures over 16 up to 21 days were subtracted from AUC values of the corresponding T20-negative samples. Individual points indicate independent experiments with cells from individual donors. Paired t-test was used for statistical analysis.

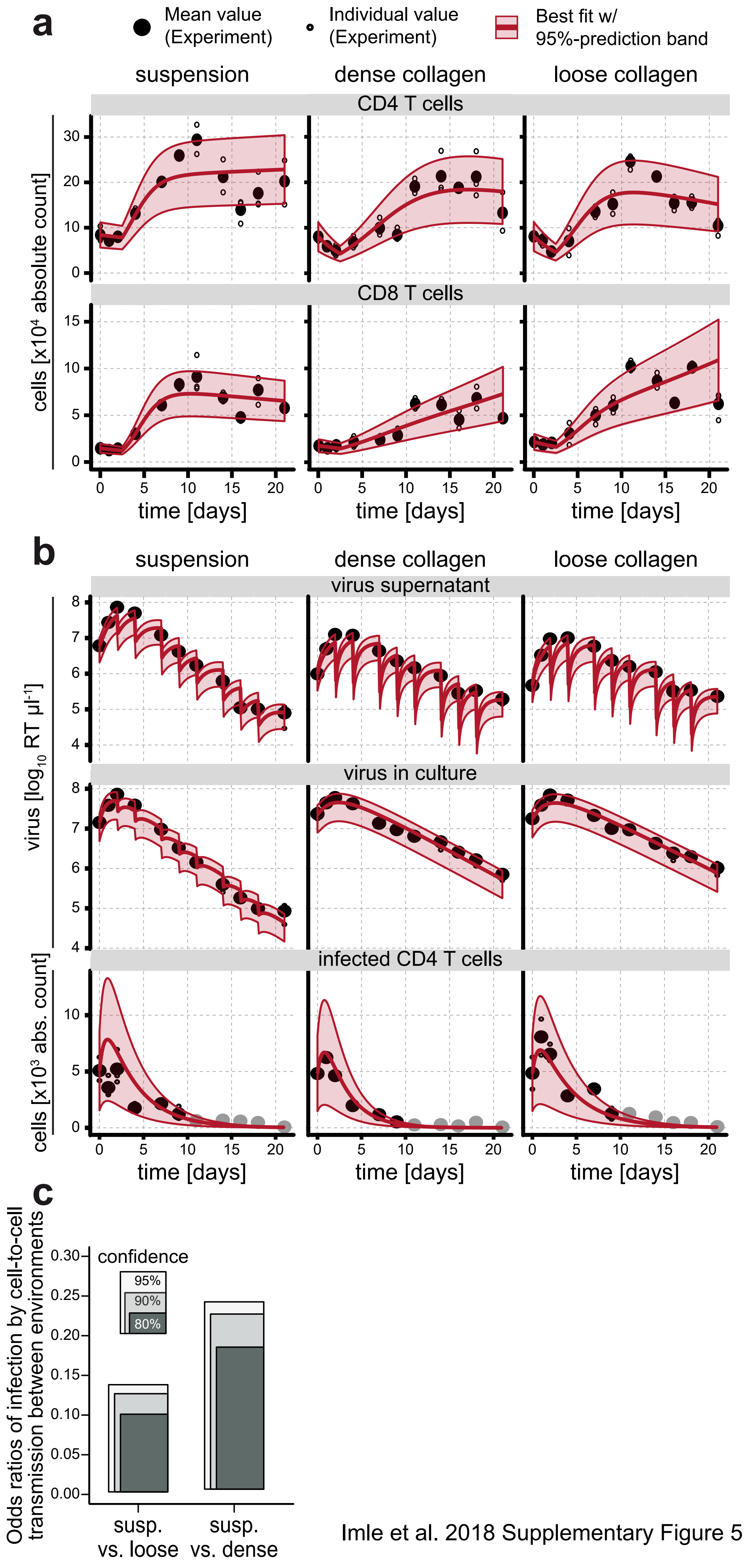

Imle et al. 2018 Supplementary Figure 5

Supplementary Figure 5. Modelling of cell proliferation and viral turnover.

(a) Modelling of absolute count data of CD4 and CD8 T cells to determine proliferation parameters. (b) Modelling of virus kinetics in T20 treated samples to determine virus production and infected cell life span. (c) Odds-ratios for infection by cell-to-cell transmission between suspension and loose (left) and suspension and dense (right) collagen based on the obtained parameter estimates for β_c and β_f (Table 1, Fig. 3e). The odds for each environment define the chance of infection by cell-to-cell transmission (P_{CC}) vs. infection by cell-free infection (P_{CF}) in this particular environment ($O=P_{CC}/P_{CF}$). The ratio between the odds for two different environments (e.g., $OR_{SL}=O_S/O_L$) then provides an estimate of how much more likely it is to be infected by cell-to-cell transmission in the one environment compared to the other. For example, an odds ratio of $O_{SD}=0.25$ as observed between suspension (S) and dense (D) collagen indicates that the chance of a cell being infected by cell-to-cell transmission is 4-times more likely in dense collagen compared to suspension. Gray-shaded areas indicate the different levels of confidence for parameter estimates.

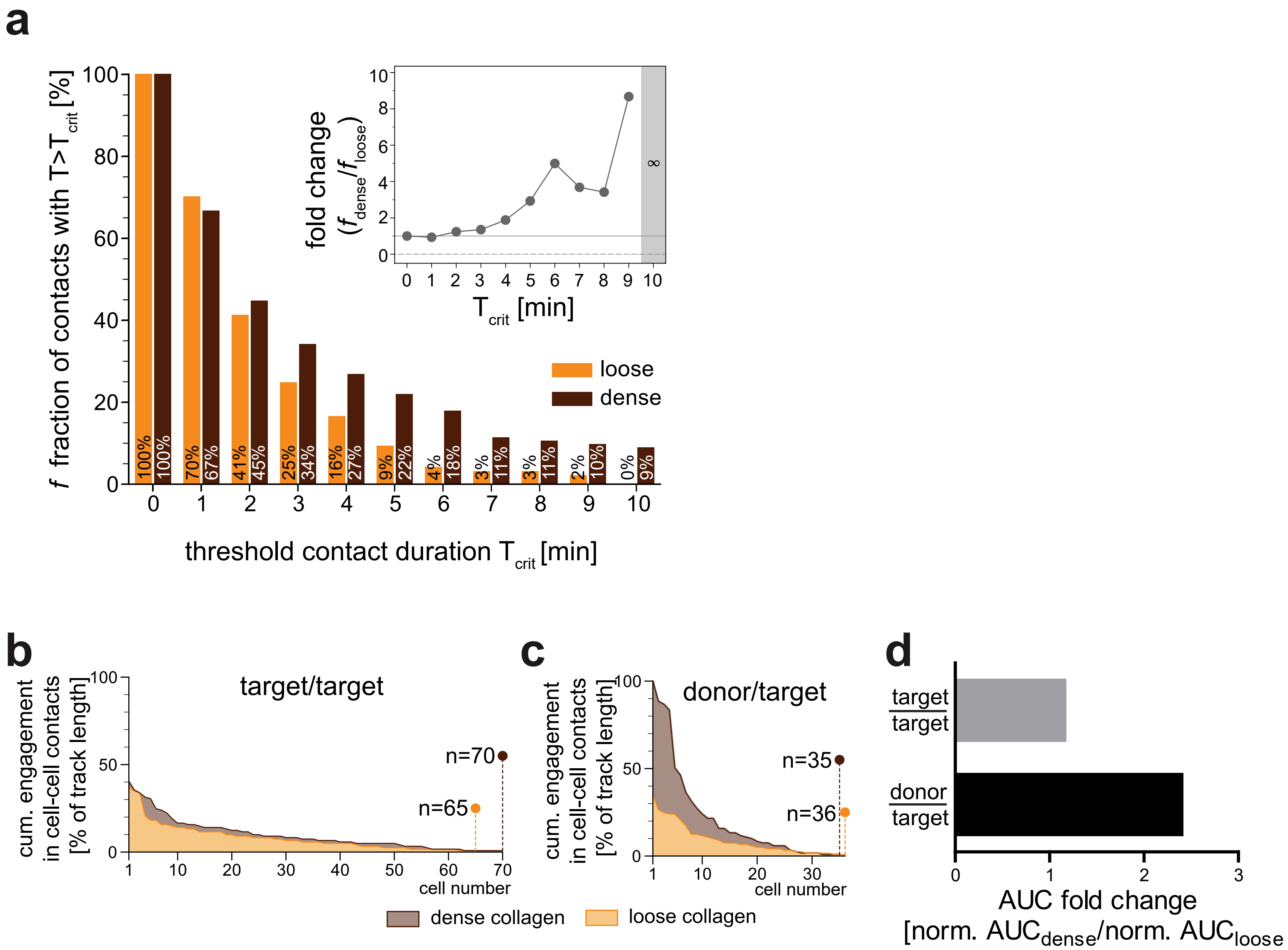

Imle et al. 2018 Supplementary Figure 6

Supplementary Figure 6. Advanced contact analysis.

(a) Fraction of cell-cell contacts exceeding a critical threshold contact duration T_{crit} for infected cell/target cell pairs. The inset shows the fold change between fraction of contacts between dense and loose collagen. (b) Cumulated target-target cell contact duration normalized to the full track duration, which is set to 100%. Brown and ochre curves depict contacts in dense and loose collagen, respectively, indicating the number of cells displaying the indicated cumulative contact duration. *n* indicates the total amount of tracks analyzed. (c) Cumulated donor-target cell contact duration displayed as in (b). (d) The difference between cumulated contacts for target/target and donor/target contacts as shown in (b) and (c) was computed as the ratio between areas under the curve (AUC) for dense and loose collagen normalized to the amount of tracks.

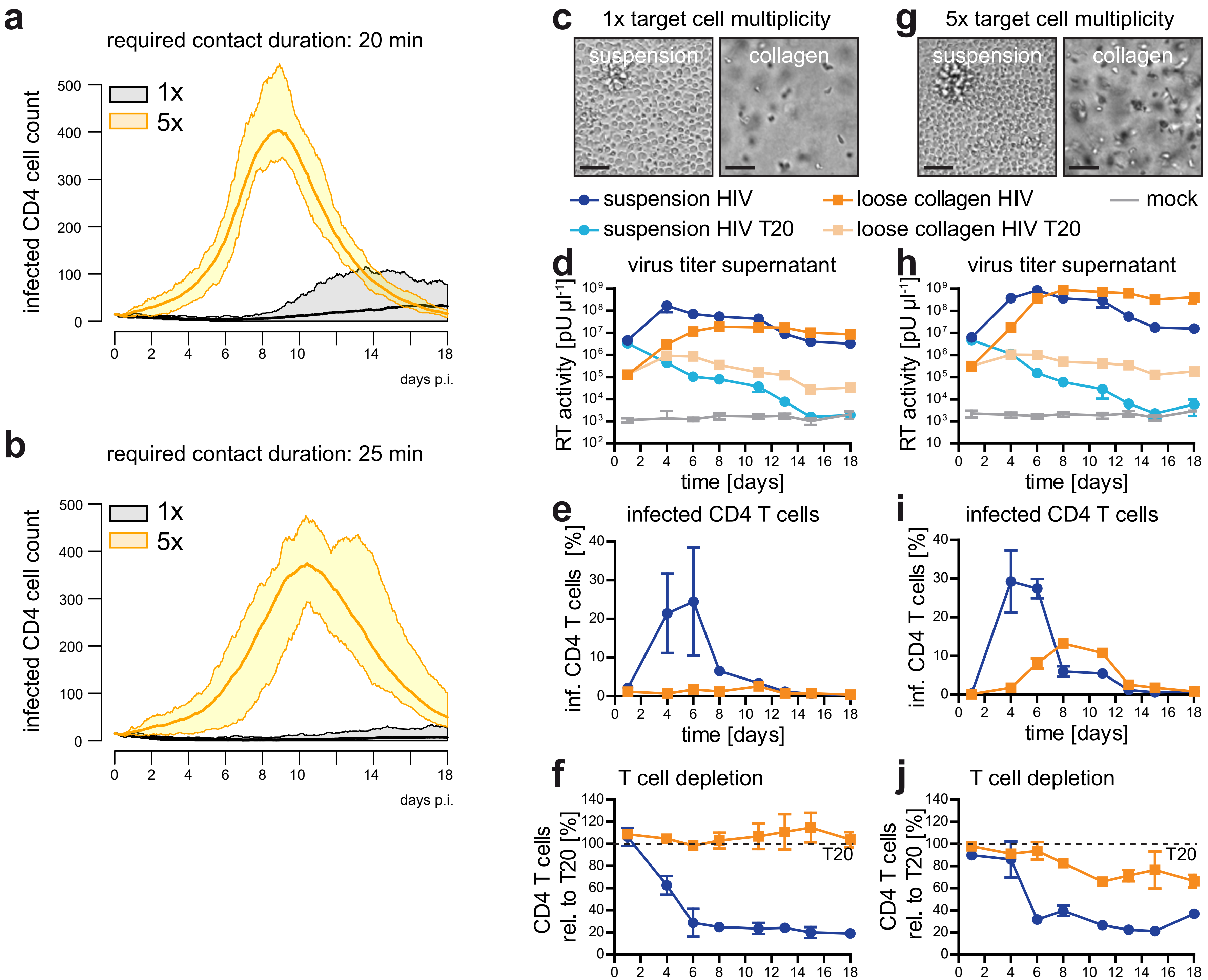

Imle et al. 2018 Figure S7

Supplementary Figure 7. CPM simulation with increased target cell concentrations confirm minimal contact duration for productive HIV-1 spread via cell-to-cell transmission.

(a, b) Simulated infection dynamics based on the CPM using the standard (1x, black) or five-fold higher (5x, orange) concentration of target cells assuming either that a contact duration of 20 (a) or 25 (b) minutes between infected and uninfected cells is necessary for cell-to-cell spread. The mean (solid lines) and the minimal and maximal number of infected cells per time point (shaded area) over 10 independent simulations per concentration are shown. (c-j) Experimental data including T20 and mock control for comparison of the infection dynamics in suspension (blue) and loose collagen (orange) using the standard (1x, c-f) and 5-fold increased (g-j) concentration of target cells. Scale bar of representative bright field images: 40 µm. (d, h) Virus titers determined from supernatants by SG-PERT. (e, i) Percentage of infected (p24+) CD4 T cells determined by FACS. (f, j) T cell depletion expressed as residual CD4 T cells relative to respective T20 control, which was set to 100% (dashed line). Mean values and standard deviations from triplicate measurement are shown.
