# Supplementary material for: Experimental and computational analyses reveal that environmental restrictions shape HIV-1 spread in 3D cultures
Source: Nat Commun. 2019 May 13;10:2144. doi: 10.1038/s41467-019-09879-3 (PMC6514199; doi:10.1038/s41467-019-09879-3)
Supplement: Supplementary file 3 — Reporting Summary [file 41467_2019_9879_MOESM3_ESM.pdf]

nature research

Corresponding author(s): Fackler-OT

Last updated by author(s): 03-27-19

Reporting Summary

Nature Research wishes to improve the reproducibility of the work that we publish. This form provides structure for consistency and transparency in reporting. For further information on Nature Research policies, see Authors & Referees and the Editorial Policy Checklist.

Statistics

For all statistical analyses, confirm that the following items are present in the figure legend, table legend, main text, or Methods section.

n/a	Confirmed	
☐	☒	The exact sample size (*n*) for each experimental group/condition, given as a discrete number and unit of measurement
☐	☒	A statement on whether measurements were taken from distinct samples or whether the same sample was measured repeatedly
☐	☒	The statistical test(s) used AND whether they are one- or two-sided<br>*Only common tests should be described solely by name; describe more complex techniques in the Methods section.*
☐	☒	A description of all covariates tested
☐	☒	A description of any assumptions or corrections, such as tests of normality and adjustment for multiple comparisons
☐	☒	A full description of the statistical parameters including central tendency (e.g. means) or other basic estimates (e.g. regression coefficient) AND variation (e.g. standard deviation) or associated estimates of uncertainty (e.g. confidence intervals)
☐	☒	For null hypothesis testing, the test statistic (e.g. *F*, *t*, *r*) with confidence intervals, effect sizes, degrees of freedom and *P* value noted<br>*Give P values as exact values whenever suitable.*
☒	☐	For Bayesian analysis, information on the choice of priors and Markov chain Monte Carlo settings
☒	☐	For hierarchical and complex designs, identification of the appropriate level for tests and full reporting of outcomes
☒	☐	Estimates of effect sizes (e.g. Cohen's *d*, Pearson's *r*), indicating how they were calculated

Our web collection on statistics for biologists contains articles on many of the points above.

Software and code

Policy information about availability of computer code

Data collection	Flow cytomertry data were collected using the FACSuite software from BD. Real time wide field microscopy data were collected using Nikon Imaging Software (NISElements). Virus titer data using SG-PERT method was collected using CFX Manager by BioRad. Spinning disc data was collected using Volocity software by Perkin Elmer. Confocal reflection data was acquired using Leica LAS AF Lite software.
Data analysis	Statistical evaluation and data presentation was performed using GraphPad Prism 5 or Excel. Ilastic (www.ilastik.org) and Image J (https://imagej.nih.gov/ij/) with MaMut plugin were used to analyze, segment and track imaging data of cell motility. Flow cytometry data was analyzed using Flow Jo software.

For manuscripts utilizing custom algorithms or software that are central to the research but not yet described in published literature, software must be made available to editors/reviewers. We strongly encourage code deposition in a community repository (e.g. GitHub). See the Nature Research guidelines for submitting code & software for further information.

Data

Policy information about availability of data

All manuscripts must include a data availability statement. This statement should provide the following information, where applicable:

- Accession codes, unique identifiers, or web links for publicly available datasets
- A list of figures that have associated raw data
- A description of any restrictions on data availability

The data supporting the findings of this study are available within the article, in supplementary files, and upon reasonable request from the corresponding author.

Field-specific reporting

Please select the one below that is the best fit for your research. If you are not sure, read the appropriate sections before making your selection.

☒ Life sciences ☐ Behavioural & social sciences ☐ Ecological, evolutionary & environmental sciences

For a reference copy of the document with all sections, see nature.com/documents/nr-reporting-summary-flat.pdf

Life sciences study design

All studies must disclose on these points even when the disclosure is negative.

Sample size	No statistical method was used to predetermine the sample size. Sample size was chosen based on previous experience and standards in the field. Sample size and number of independent experiments are stated in the figure legend or in the Methods section. Three to more independent results were used to perform statistical analyses.
Data exclusions	No data is excluded if the experiments were successfully performed.
Replication	All experiments were reliably reproduced.
Randomization	Data was not randomized
Blinding	Data was not blinded for analysis

Reporting for specific materials, systems and methods

We require information from authors about some types of materials, experimental systems and methods used in many studies. Here, indicate whether each material, system or method listed is relevant to your study. If you are not sure if a list item applies to your research, read the appropriate section before selecting a response.

Materials & experimental systems

n/a	Involved in the study
☐	☒ Antibodies
☐	☒ Eukaryotic cell lines
☒	☐ Palaeontology
☒	☐ Animals and other organisms
☒	☐ Human research participants
☒	☐ Clinical data

Methods

n/a	Involved in the study
☒	☐ ChIP-seq
☐	☒ Flow cytometry
☒	☐ MRI-based neuroimaging

Antibodies

Antibodies used	anti-CD3 (clone HIT3a against CD3ε; clone OKT3: cell line supernatant, ATCC), anti-CD3-PE (biolegend), anti-CD8-PE Vio770 (Miltenyi Biotec), anti HIV p24 (KC57, Beckman Coulter)
Validation	the antibodies were not validated by ourselves.

Eukaryotic cell lines

Policy information about cell lines

Cell line source(s)	Hek293T and OKT3 cell lines were originally obtained from ATCC. TZM-bl cells were obtained from NIH AIDS repository
Authentication	Cell lines were not authenticated by ourselves.
Mycoplasma contamination	All cell lines were routinely tested for mycoplasma and no contamination was revealed.
Commonly misidentified lines (See ICLAC register)	No commonly misidentified cell line was used.

Flow Cytometry

Plots

Confirm that:

☒ The axis labels state the marker and fluorochrome used (e.g. CD4-FITC).

☒ The axis scales are clearly visible. Include numbers along axes only for bottom left plot of group (a 'group' is an analysis of identical markers).

☒ All plots are contour plots with outliers or pseudocolor plots.

☒ A numerical value for number of cells or percentage (with statistics) is provided.

Methodology

Sample preparation	sample preparation of PBMC for flow cytometry is described in detail in Methods section
Instrument	FACSVerse (BD)
Software	data was collected with FACSuite (BD) and anaysed with FlowJo software (FlowJo LLC)
Cell population abundance	only analysis but no sorting was performed
Gating strategy	illustrated in Supplementary Figure 1, gating on cells by FSC/SCC, exclusion of dead cells by fixable viability dye (ebioscience), CD4 cells (CD3+, CD8-, p24 positive CD4 cells

☒ Tick this box to confirm that a figure exemplifying the gating strategy is provided in the Supplementary Information.
