# Supplementary material for: Experimental and computational analyses reveal that environmental restrictions shape HIV-1 spread in 3D cultures
Source: Nat Commun. 2019 May 13;10:2144. doi: 10.1038/s41467-019-09879-3 (PMC6514199; doi:10.1038/s41467-019-09879-3)
Supplement: Supplementary file 4 — Description of Additional Supplementary Files [file 41467_2019_9879_MOESM4_ESM.pdf]

Description of Additional Supplementary Files

File Name: Supplementary Movie 1
Description: **Migration of uninfected CD4 T cells in dense collagen.** Mock infected CD4 T cells were stained with PKH cell dye and embedded in dense collagen. Representative cells were imaged in bright-field mode for every 30 sec intervals, segmented and tracked for 25 min with tracks indicating individual cells. Scale bar 50 µm.

File Name: Supplementary Movie 2
Description: **Migration of uninfected CD4 T cells in loose collagen.** Mock infected CD4 T cells were stained with PKH cell dye and embedded in loose collagen. Representative cells were imaged in bright-field for every 30 sec intervals, segmented and tracked for 25 min with tracks indicating individual cells. Scale bar 50 µm.

File Name: Supplementary Movie 3
Description: **Contacts of target CD4 T cells in co-culture with HIV-1 infected cells.** Representative purified uninfected CD4 cells and purified HIV-1 infected cells were stained red and green with PKH cell dyes, respectively, mixed and embedded in dense (left) and loose (loose) collagen. Cells were imaged every 30 s, segmented and tracked. Green: HIV-1 infected cell, red: target CD4 T cell. Contacts are indicated by an arrow head. Scale bar: 20 µm.

File Name: Supplementary Movie 4
Description: **CPM simulation of target CD4 T cells in co-culture with HIV-1 infected cells in loose collagen.** Simulated cell dynamics in the CPM with the parameterization as obtained from adaptation of individual cell motilities to the live-cell imaging data. Recapitulating experimental conditions, a 1:1 ratio with a total number of 150 target (red) and 150 infected cells (green) is shown not allowing for infections. Collagen is indicated in grey. The simulated time period covers 1h in real time.
