# Supplementary material for: Experimental and computational analyses reveal that environmental restrictions shape HIV-1 spread in 3D cultures
Source: Nat Commun. 2019 May 13;10:2144. doi: 10.1038/s41467-019-09879-3 (PMC6514199; doi:10.1038/s41467-019-09879-3)
Supplement: Supplementary file 2 — Peer Review File [file 41467_2019_9879_MOESM2_ESM.pdf]

Editorial Note: This manuscript has been previously reviewed at another journal that is not operating a transparent peer review scheme. This document only contains reviewer comments and rebuttal letters for versions considered at Nature Communications .

Reviewers' Comments:

Reviewer #1:
None
